# Essential Oils from Seed-Depleted Infructescences of Industrial Hemp: Chemical Diversity and Biological Potential

**DOI:** 10.3390/molecules31162886

**Published:** 2026-08-18

**Authors:** Piotr Sugier, Aleksandra Nurzyńska, Małgorzata Miazga-Karska, Danuta Sugier, Radosław Kowalski, Karolina Jaros-Tsoj, Dawid Świstak, Jolanta Jaroszuk-Ściseł, Jaco Vangronsveld, Andrzej Plak, Małgorzata Wójcik

**Affiliations:** 1 Department of Botany, Mycology and Ecology, Institute of Biological Sciences, Maria Curie-Skłodowska University, Akademicka 19, 20-033 Lublin, Poland; 2 Chair and Department of Biochemistry and Biotechnology, Medical University of Lublin, 1 Chodźki Street, 20-093 Lublin, Poland; aleksandra.nurzynska@umlub.edu.pl (A.N.); malgorzata.miazga-karska@umlub.edu.pl (M.M.-K.); 3 Department of Industrial and Medicinal Plants, University of Life Sciences in Lublin, 15 Akademicka Street, 20-950 Lublin, Poland; danuta.sugier@up.lublin.pl; 4 Department of Analysis and Evaluation of Food Quality, University of Life Sciences in Lublin, 8 Skromna Street, 20-704 Lublin, Poland; radoslaw.kowalski@up.lublin.pl; 5 Department of Plant Physiology and Biophysics, Institute of Biological Sciences, Maria Curie-Skłodowska University, Akademicka 19, 20-033 Lublin, Poland; karolina.jaros-tsoj@mail.umcs.pl (K.J.-T.); dawid.swistak@mail.umcs.pl (D.Ś.); jaco.vangronsveld@mail.umcs.pl (J.V.); 6 Department of Industrial and Environmental Microbiology, Institute of Biological Sciences, Maria-Curie Skłodowska University, Akademicka 19, 20-033 Lublin, Poland; jolanta.jaroszuk-scisel@mail.umcs.pl; 7 Centre for Environmental Sciences, Hasselt University, Agoralaan Buidling D, B-3590 Diepenbeek, Belgium; 8 Department of Geology, Soil Science and Geoinformation, Maria Curie-Skłodowska University, al. Kraśnicka 2D, 20-718 Lublin, Poland; andrzej.plak@mail.umcs.pl

**Keywords:** *Cannabis sativa*, *Cutibacterium acnes*, cytotoxicity, haemolytic activity, antibacterial properties

## Abstract

Industrial hemp is a chemically rich plant increasingly explored within sustainable production systems aimed at the full utilization of all plant fractions. While hemp inflorescences have been extensively investigated, the biological potential of seed-depleted infructescences remains largely underexplored. This study compared essential oils (EOs) distilled from inflorescences and seed-depleted infructescences of hemp (*Cannabis sativa* L. cv. Futura 75). Their chemical composition was determined by gas chromatography–mass spectrometry (GC–MS), and their cytotoxicity, hemocompatibility, effects on blood coagulation, and antibacterial activity against a panel of 11 Gram-positive, Gram-negative, and microaerophilic bacterial strains were evaluated. The major EO constituents included α-Pinene, (E)-Caryophyllene, Myrcene, α-Humulene, Caryophyllene oxide, and Cannabidiol. EOs obtained from seed-depleted infructescences exhibited distinct chemical profiles, low cytotoxicity toward BJ human skin fibroblasts, minimal haemolytic activity, no significant effects on blood coagulation, and, in most cases, stronger antibacterial activity than inflorescence-derived EOs. Particularly high activity was observed against skin- and oral-associated bacteria, including *Cutibacterium acnes* and *Streptococcus* species. These findings demonstrate that seed-depleted infructescences represent underutilized post-harvest biomass and a valuable and sustainable source of biologically active EOs, supporting their further investigation for potential pharmaceutical, cosmetic, and oral healthcare applications.

## 1. Introduction

Industrial hemp (*Cannabis sativa* L. subsp. *sativa*) has been valued as a versatile and high-yield crop, and interest in hemp-derived products continues to expand steadily [1,2]. Its stems provide fibre, hurd, and other components used in textile manufacturing, paper production, construction materials, and biocomposites for a wide range of applications. Hemp seeds are an important source of dietary fibre, proteins, and especially oil, which finds applications in the pharmaceutical, cosmetic, and industrial sectors. Inflorescences contain numerous valuable metabolites, including fatty acids, flavonoids, amino acids, terpenes, terpenophenolics, sugars, and hydrocarbons [3]. Hemp seeds are also a valuable source of natural ingredients for functional foods [4]. As a result of hemp cultivation and processing, substantial quantities of biomass remain underutilised, including inflorescences, seed-depleted infructescences, and residues generated during extraction procedures. Researchers emphasise the importance of enhancing the sustainability of hemp cultivation systems. Agronomic practices should therefore be optimised not only to improve productivity but also to maximise resource efficiency through the comprehensive use of all plant fractions, particularly the diverse bioactive compounds present in processing residues [1,3,5]. Several hemp-derived residues have shown potential as botanical insecticides, miticides, and repellents for pest management in organic farming [6,7]. Furthermore, the sustainable utilization of seed-depleted infructescences remaining after seed harvest remains insufficiently explored and warrants further investigation.

Hemp inflorescences have attracted considerable interest due to their rich chemical composition and wide range of potential applications [2,8,9,10]. Among the valuable products obtained from this plant are essential oils (EOs). The chemical composition of hemp Eos is highly variable, with up to approximately one hundred compounds identified to date [9,11,12,13,14,15,16]. Sesquiterpenes generally constitute the predominant fraction of hemp EO, exceeding the proportion of monoterpenes [17], although cannabinoids have also been reported [12,15,16,18]. Among the sesquiterpenes, the major constituents include β-Caryophyllene, Caryophyllene oxide, α-Humulene, and Limonene, whereas the predominant monoterpenes include α-Pinene, β-Myrcene, Terpinolene, and β-Pinene [12,13,14,15,16,17,18,19]. The most frequently reported cannabinoids are Cannabidiol (CBD) and Cannabidiolic acid (CBDA) [12,16,19]. Due to this complex chemical composition, hemp EOs exhibit antibacterial [11,12,19,20,21,22] and antifungal activities [20,23,24], antioxidant potential [18,21,22], antidiabetic properties [23], and anti-inflammatory activity [7,24,25]. In addition to these activities, preclinical studies have suggested that hemp EOs possess analgesic, neuroprotective, and anticancer potential, highlighting their promising therapeutic applications [26,27,28].

Application of hemp EOs as tonic ingredients in beverages, flavouring agents, and nutraceutical components of certified foods has recently been proposed [29]. They are also characterized by insecticidal and repellent activities [25,30,31], and have been proposed as effective agents for insect control [6]. A comparative study of Italian hemp varieties, including Futura 75, the subject of our study, demonstrated antifungal activity, suggesting potential applications of hemp EOs in dermatological and cosmetic formulations [24]. These findings also justify investigating the activity of hemp EOs against skin-associated bacterial pathogens, many of which have not yet been evaluated.

The yield and composition of individual secondary metabolites in hemp EO, as well as their chemical variability and biological activity, depend on many factors, including the sex of the inflorescences [25], genetic diversity of cultivars [13,14,20,24,32,33,34], environmental conditions [13,18,31,33,34], plant density and mineral fertilization [35], weather conditions [34], the developmental stage of the hemp infructescence [2,6,13,14,18,36,37], the phenological stage of the plant [14,38,39], and harvest time [34]. Moreover, at the laboratory level, the yield and composition of hemp Eos are also influenced by the type of plant material (fresh or dried) [6,40,41], drying technique, and extraction procedure [5,17,18,20,31,40,42,43]. In addition, a recent study demonstrated that the position of the raw material within the inflorescence can substantially affect both the yield and chemical composition of the final product [16]. Consequently, selecting the appropriate harvest stage and plant material is crucial for obtaining hemp EOs with distinct chemical compositions and, consequently, different biological properties and potential industrial applications [2]. Combining different plant parts and developmental stages enables the generation of a broader diversity of EO chemical profiles within a single growing season, thereby increasing their biological and application potential.

Considering the entire range of factors mentioned above, the extensive literature reports substantial variability in the chemical composition of hemp EOs. Although an increasing number of studies have investigated the use of seed-depleted infructescences (chaff), which retain oil trichomes even at the end of the growing season [16,33], their secondary metabolite composition and biological activity have not yet been directly compared with those of inflorescences, which remain the plant material most frequently investigated, with most studies focusing on the upper 20–30 cm of the inflorescence [6,7,15,32]. Moreover, such a comparison has not been performed within a single field experiment conducted during one growing season, thereby eliminating the influence of environmental and agronomic variables such as soil properties, weather conditions, and crop management practices.

Therefore, the present study was based on the hypothesis that the chemical composition and biological activity of hemp EOs vary depending on the type of plant material, its developmental stage, and its position within the inflorescence or seed-depleted infructescence. Accordingly, the objectives were: (i) to characterize the chemical profiles of EOs obtained from the upper and lower parts of inflorescences and seed-depleted infructescences; (ii) to evaluate their cytotoxic and antibacterial activities, effects on fibroblast metabolic activity, and haemolytic activity; and (iii) to evaluate their effect on blood coagulation. Particular attention was paid to essential oils obtained from seed-depleted infructescences as a potentially valuable and underutilized source of biologically active compounds. A better understanding of the chemical composition and biological properties of these EOs, derived from different raw materials, particularly their activity against oral and skin-associated bacteria, may facilitate their future application in antimicrobial topical formulations and other value-added products.

## 2. Results and Discussion

### 2.1. Characteristics and Differentiation of the Yield and Chemical Composition of Essential Oils

Two-way ANOVA revealed significant effects of raw material type (F = 6.84, *p* = 0.031) and position within the inflorescence/infructescence (F = 30.99, *p* < 0.0001) on the EO content expressed as a percentage of dry mass, whereas their interaction was not significant (F = 2.09, *p* = 0.186) (Table 1). EO content ranged from 0.265% in LFL to 0.288% in UFL and from 0.272% in LFR to 0.310% in UFR. The EO content was significantly higher in the upper than in the lower part of the infructescences, whereas comparable values were recorded for UFL and UFR. Notably, the comparable EO content of UFL and UFR supports the potential use of seed-depleted infructescences, an underutilized post-harvest material, as an additional source of EOs in hemp cultivated for seed, as previously suggested [16].

The EOs distilled from plant material collected at different developmental stages and positions showed distinct chemical profiles (Table 2). A total of 80 compounds were tentatively identified in both UFL and LFL oils, compared with 71 and 68 compounds in UFR and LFR oils, respectively. These constituents accounted for 98.85%, 95.94%, 94.21%, and 93.31% of the total composition of UFL, LFL, UFR, and LFR oils, respectively. The number of identified EO constituents exceeded that previously reported for Futura 75 EOs from different cultivation sites in Italy (55–74 compounds) [15] and in other studies of this cultivar (23–40 compounds) [18,21,24]. However, such comparisons should be interpreted cautiously, as the number of identified constituents may be influenced not only by environmental and agronomic conditions but also by differences in plant material, EO isolation, and analytical procedures.

At the class level, the inflorescence-derived oils were dominated by sesquiterpenes, with sesquiterpene hydrocarbons and oxygenated sesquiterpenes together accounting for 79.99% and 80.44% of UFL and LFL, respectively. In contrast, the infructescence-derived oils contained markedly higher proportions of monoterpene hydrocarbons, reaching 40.69% in UFR and 30.95% in LFR. Cannabinoids represented 7.75%, 8.27%, 3.17%, and 8.07% of the identified constituents in UFL, LFL, UFR, and LFR, respectively. The distribution of the major chemical classes is summarized in the final rows of Table 2. The broad chemical diversity observed in Futura 75 EOs was also evident when compared with other industrial hemp cultivars, for which 38–77 constituents have been reported depending on the cultivar and plant material used [11,19,22,40]. These comparisons further illustrate the substantial variability in hemp EO composition across cultivars and experimental conditions [44].

**Table 2:** Chemical composition of essential oils from hemp inflorescences and infructescences. UFL—flowers, bracts and leaves from the upper part of the inflorescence, LFL—flowers, bracts and leaves from the lower part of the inflorescence, UFR—bracts and leaves from the upper part of the infructescence, LFR—bracts and leaves from the lower part of the infructescence.

No.	Compound	RI_exp_	RI_Lit_	UFL		LFL		UFR		LFR	
				[%]	±SD	[%]	±SD	[%]	±SD	[%]	±SD
1	Heptanal	904.0	902	–	–	–	–	0.31	±0.019	0.23	±0.014
2	*α*-Thujene	937.6	930	0.18	±0.006	0.01	±0.001	0.15	±0.005	0.13	±0.012
3	*α*-Pinene	945.4	939	4.52	±0.219	3.10	±0.138	17.90	±0.458	14.75	±0.122
4	Camphene	962.2	954	0.01	±0.001	0.01	±0.001	0.42	±0.006	0.33	±0.005
5	Sabinene	985.8	975	0.19	±0.01	0.01	±0.001	0.12	±0.004	0.13	±0.009
6	*β*-Pinene	991.2	979	1.60	±0.076	1.30	±0.076	4.96	±0.201	4.04	±0.081
7	1-Octen-3-ol	994.2	978	0.01	±0.001	0.01	±0.001	–	–	–	–
8	6-Methyl-5-hepten-2-one	999.3	986	0.01	±0.001	0.01	±0.001	–	–	–	–
9	Myrcene	1002.2	991	1.65	±0.083	1.29	±0.076	8.10	±0.268	5.61	±0.109
10	*α*-Phellandrene	1017.1	1003	0.01	±0.001	0.01	±0.001	0.17	±0.011	0.15	±0.006
11	*δ*-3-Carene	1019.2	1010	0.05	±0.003	0.01	±0.001	0.56	±0.020	0.25	±0.003
12	*α*-Terpinene	1026.4	1017	0.01	±0.001	0.01	±0.001	0.15	±0.002	0.16	±0.012
13	*p*-Cymene	1033.5	1025	0.01	±0.001	0.01	±0.001	0.12	±0.007	0.17	±0.017
14	Limonene	1037.4	1029	0.17	±0.009	0.09	±0.003	1.80	±0.095	0.89	±0.020
15	*β*-Phellandrene	1038.9	1030	0.01	±0.001	0.01	±0.001	0.41	±0.049	0.36	±0.013
16	1.8-Cineole	1040.6	1031	0.46	±0.019	0.11	±0.008	0.22	±0.021	0.57	±0.014
17	(Z)-*β*-Ocimene	1042.9	1037	0.09	±0.007	0.05	±0.002	0.57	±0.017	0.25	±0.004
18	(E)-*β*-Ocimene	1052.4	1050	0.24	±0.013	0.11	±0.008	2.64	±0.065	1.08	±0.023
19	*γ*-Terpinene	1063.4	1060	0.01	±0.001	0.08	±0.006	0.22	±0.026	0.26	±0.008
20	cis-Sabinene hydrate	1075.3	1070	0.01	±0.001	0.01	±0.001	0.13	±0.017	0.14	±0.017
21	Terpinolene	1088.2	1089	0.24	±0.006	0.01	±0.001	2.30	±0.008	2.32	±0.044
22	*p*-Cymenene	1093.3	1091	0.01	±0.001	0.01	±0.001	0.10	±0.012	0.06	±0.003
23	Linalool	1102.2	1097	0.39	±0.018	0.01	±0.001	0.31	±0.016	0.26	±0.005
24	trans-Sabinene hydrate	1104.4	1098	0.01	±0.001	0.14	±0.001	0.06	±0.002	0.08	±0.007
25	exo-Fenchol	1122.0	1122	–	–	–	–	0.19	±0.003	0.05	±0.001
26	endo-Fenchol	1125.3	1117	0.14	±0.011	0.17	±0.011	–	–	–	–
27	trans-*p*-Mentha-2.8-dien-1-ol	1130.5	1123	0.01	±0.001	0.01	±0.001	–	–	–	–
28	cis-*p*-Menth-2-en-1-ol	1132.9	1122	0.08	±0.003	0.17	±0.003	–	–	–	–
29	trans-Pinene hydrate	1136.7	1123	0.15	±0.009	0.01	±0.009	0.20	±0.021	0.11	±0.014
30	trans-Pinocarveol	1150.7	1139	0.19	±0.013	0.10	±0.013	0.52	±0.031	0.61	±0.016
31	cis-Pinene hydrate	1156.1	1144	0.35	±0.022	0.01	±0.022	–	–	–	–
32	*β*-Pinene oxide	1158.0	1159	–	–	–	–	0.24	±0.018	0.07	±0.000
33	*p*-Mentha-1.5-dien-8-ol	1179.2	1170	0.01	±0.001	0.01	±0.001	–	–	–	–
34	Borneol	1185.1	1169	0.04	±0.003	0.04	±0.003	0.12	±0.005	0.08	±0.005
35	Terpinen-4-ol	1191.9	1177	0.18	±0.008	0.13	±0.008	0.86	±0.041	0.87	±0.041
36	Myrtenol	1199.0	1196	–	–	–	–	0.09	±0.004	0.12	±0.007
37	*p*-Cymen-8-ol	1201.7	1183	0.01	±0.001	0.01	±0.001	0.14	±0.008	0.11	±0.008
38	*α*-Terpineol	1209.8	1189	0.01	±0.001	0.01	±0.001	0.19	±0.014	0.11	±0.006
39	Safranal	1214.2	1197	0.01	±0.001	0.01	±0.001	–	–	–	–
40	trans-Piperitol	1224.1	1208	0.01	±0.001	0.01	±0.001	–	–	–	–
41	trans-Chrysanthenyl acetate	1227.0	1238	–	–	–	–	0.10	±0.007	0.11	±0.008
42	*α*-Ylangene	1381.2	1375	0.01	±0.001	0.31	±0.022	0.07	±0.005	0.07	±0.002
43	*α*-Copaene	1387.5	1377	0.37	±0.008	0.55	±0.013	–	–	–	–
44	7-epi-Sesquithujene	1415.7	1391	0.01	±0.001	0.01	±0.001	–	–	–	–
45	(Z)-Caryophyllene	1418.9	1409	6.02	±0.126	12.87	±0.338	0.32	±0.007	0.34	±0.016
46	*α*-Gurjunene	1421.8	1410	0.58	±0.007	6.06	±0.135	–	–	–	–
47	*α*-cis-Bergamotene	1428.8	1413	4.64	±0.305	0.01	±0.001	0.16	±0.020	0.26	±0.007
48	(E)-Caryophyllene	1438.2	1419	5.22	±0.219	5.43	±0.226	14.31	±0.181	12.46	±0.139
49	(Z)-*β*-Farnesene	1443.4	1440	0.01	±0.001	1.74	±0.08	–	–	–	–
50	*α*-trans-Bergamotene	1451.1	1435	0.43	±0.008	0.84	±0.029	1.13	±0.029	1.66	±0.007
51	Aromadendrene	1460.6	1441	0.01	±0.001	0.24	±0.011	–	–	–	–
52	(E)-*β*-Farnesene	1473.2	1454	8.01	±0.411	6.24	±0.352	1.12	±0.030	1.58	±0.032
53	*α*-Humulene	1478.5	1455	2.90	±0.033	2.08	±0.033	5.47	±0.056	4.72	±0.059
54	allo-Aromadendrene	1483.2	1460	0.31	±0.014	0.44	±0.014	0.38	±0.029	0.51	±0.049
55	*δ*-Selinene	1491.0	1493	–	–	–	–	1.17	±0.064	1.02	±0.019
56	*γ*-Muurolene	1501.3	1480	0.30	±0.022	0.39	±0.022	0.12	±0.014	–	–
57	*α*-Amorphene	1504.1	1485	1.06	±0.037	0.50	±0.037	0.05	±0.005	–	–
58	*α*-Bulnesene	1505.0	1510	–	–	–	–	0.28	±0.003	0.20	±0.013
59	*β*-Chamigrene	1508.2	1478	0.01	±0.001	1.56	±0.065	–	–	–	–
60	*β*-Selinene	1513.0	1490	1.17	±0.045	2.06	±0.059	0.40	±0.010	0.41	±0.000
61	*γ*-Cadinene	1515.0	1514	–	–	–	–	0.17	±0.023	0.13	±0.018
62	Valencene	1515.9	1496	0.01	±0.001	0.01	±0.001	0.24	±0.010	0.17	±0.010
63	*α*-Selinene	1517.7	1498	1.21	±0.037	0.91	±0.031	0.83	±0.058	0.70	±0.011
64	*δ*-Cadinene	1521.0	1523	–	–	–	–	0.19	±0.015	–	–
65	*δ*-Amorphene	1525.1	1512	0.70	±0.037	0.95	±0.053	–	–	–	–
66	(Z)-*γ*-Bisabolene	1528.8	1515	1.20	±0.065	0.47	±0.025	0.13	±0.009	0.15	±0.008
67	*β*-Curcumene	1530.3	1516	0.01	±0.001	0.33	±0.016	0.15	±0.000	0.16	±0.008
68	(E)-*γ*-Bisabolene	1535.0	1531	–	–	–	–	0.54	±0.035	0.37	±0.017
69	7-epi-*α*-Selinene	1539.9	1522	0.33	±0.014	0.62	±0.014	–	–	–	–
70	*β*-Sesquiphellandrene	1543.0	1523	0.64	±0.034	0.43	±0.025	0.15	±0.010	0.14	±0.004
71	Selina-3.7(11)-diene	1551.6	1547	5.03	±0.141	4.04	±0.124	1.07	±0.017	0.88	±0.040
72	Caryophyllenyl alcohol	1569.0	1572	–	–	–	–	0.14	±0.012	0.23	±0.017
73	trans-Sesquisabinene hydrate	1570.2	1579	0.03	±0.001	0.90	±0.045	–	–	–	–
74	(E)-Nerolidol	1575.4	1563	1.23	±0.062	0.43	±0.022	0.53	±0.025	0.58	±0.026
75	Caryophyllene oxide	1596.0	1583	12.89	±0.314	8.89	±0.323	7.85	±0.169	9.99	±0.072
76	Globulol	1598.0	1585	–	–	–	–	0.24	±0.003	0.34	±0.024
77	Viridiflorol	1609.0	1593	–	–	–	–	0.28	±0.014	0.66	±0.029
78	allo-alloaromadendrene epoxide	1620.0	1641	–	–	–	–	0.61	±0.007	0.60	±0.043
79	Humulene epoxide II	1627.9	1608	5.33	±0.116	4.84	±0.128	2.43	±0.016	3.13	±0.228
80	Selina-3.11-dien-6*α*-ol	1651.0	1644	–	–	–	–	1.02	±0.084	1.02	±0.053
81	Caryophylla-4(14).8(15)-dien-5*α*-ol	1655.7	1641	3.70	±0.112	2.33	±0.105	0.40	±0.005	0.50	±0.037
82	Caryophylla-4(14).8(15)-dien-5*β*-ol	1660.6	1641	4.81	±0.121	4.17	±0.121	2.22	±0.120	3.76	±0.105
83	Bulnesol	1663.0	1672	–	–	–	–	0.15	±0.011	0.29	±0.015
84	14-hydroxy-9-epi-(Z)-Caryophyllene	1671.5	1662	3.97	±0.124	5.71	±0.128	0.62	±0.031	1.18	±0.081
85	Junicedranol	1679.0	1693	–	–	–	–	0.17	±0.012	0.26	±0.017
86	14-hydroxy-9-epi-(E)-Caryophyllene	1699.8	1670	6.72	±0.319	3.06	±0.152	1.07	±0.102	1.95	±0.075
87	*α*-Bisabolol	1711.6	1686	0.73	±0.052	0.58	±0.034	0.14	±0.013	0.08	±0.005
88	Eudesm-7(11)-en-4-ol	1724.1	1700	0.39	±0.018	0.44	±0.014	0.25	±0.014	0.27	±0.016
89	Cembrene	1959.5	1939	0.01	±0.001	0.04	±0.002	–	–	–	–
90	(3E)-Cembrene A	1991.1	1949	0.01	±0.001	0.04	±0.002	–	–	–	–
91	(3Z)-Cembrene A	2012.3	1967	0.01	±0.001	0.05	±0.002	–	–	–	–
92	Cannabidivarin (CBDV)	2274.3	2193– 2208	0.01	±0.001	0.03	±0.002	–	–	–	–
93	Cannabichromene (CBC)	2373.4	2373	0.01	±0.001	0.04	±0.002	–	–	–	–
94	Cannabidiol (CBD)	2419.6	2369– 2434	7.70	±0.348	8.09	±0.348	3.17	0.145	8.07	0.079
95	Cannabidiol derivative	2428.9		0.01	±0.001	0.05	±0.002	–	–	–	–
96	Δ8-Tetrahydrocannabinol	2469.5	2415–2450	0.01	±0.001	0.01	±0.001	–	–	–	–
97	Δ9-Tetrahydrocannabinol	2503.3	2430–2450	0.01	±0.001	0.05	±0.002	–	–	–	–
	Monoterpene hydrocarbons			9.00		6.12		40.69		30.95	
	Oxygenated monoterpenes			2.06		0.96		3.38		3.29	
	Sesquiterpene hydrocarbons			40.19		49.09		28.44		25.92	
	Oxygenated sesquiterpenes			39.80		31.35		18.14		24.84	
	Diterpene hydrocarbons			0.03		0.13		–		–	
	Cannabinoids			7.75		8.27		3.17		8.07	
	Other compounds			0.02		0.02		0.39		0.25	
	Total identified			98.85	–	95.94	–	94.21	–	93.31	–

RIexp, experimental retention index; RIlit, literature retention index [17,45]. All compounds were tentatively identified by comparison of EI mass spectra and retention indices; no analyte-specific authentic standards were used. Chemical-class totals were calculated by summing the mean relative contents of the compounds assigned to each class.

The monoterpene-rich profile of UFR and LFR is partly consistent with previous studies on Futura 75. Palmieri et al. [18] reported α-Pinene contents of 16–17% (together with high amounts of β-Caryophyllene, Humulene, and Caryophyllene oxide), comparable to the 17.90% and 14.75% found by us in UFR and LFR, respectively, whereas Ascrizzi et al. [15] reported lower and site-dependent levels of α-Pinene (4.7–9.4%), β-pinene (1.2–2.6%), and Myrcene (1.4–6.4%). The relatively low monoterpene content in UFL and LFL may reflect several, not mutually exclusive, factors, including the anatomical origin of the oil, environmental conditions, and plant developmental stage. Stalked glandular trichomes of cannabis exhibit more strongly monoterpene-dominant profiles than sessile trichomes of mature flowers and vegetative leaves [46], suggesting that differences in the proportions of these tissues may contribute to the observed EO profiles. Environmental factors, including temperature and rainfall, as well as developmental stage, have also been shown to affect the relative abundance of mono- and sesquiterpenes in hemp EOs [14,15]. The sesquiterpene profile further differentiated the EOs. UFR and LFR contained higher levels of (E)-Caryophyllene and α-Humulene than UFL and LFL, whereas UFL and particularly LFL were enriched in (Z)-Caryophyllene. This finding is noteworthy because previously reported Futura 75 EOs are typically dominated by β- or (E)-Caryophyllene, while (Z)-Caryophyllene usually occurs only at low levels. For example, Ascrizzi et al. [15] reported only 0.3–0.5% (Z)-Caryophyllene, compared with 19.1–27.1% β-Caryophyllene. In the present study, (Z)-Caryophyllene accounted for 6.02% of UFL and 12.87% of LFL, but only 0.32% and 0.34% of UFR and LFR, respectively. Therefore, the high (Z)-Caryophyllene content of LFL is one of the most distinctive compositional features of EO in the present study and may indicate a different biochemical, developmental or processing-related profile.

The CBD content was relatively high in UFL, LFL, and LFR (7.70%, 8.09%, and 8.07%, respectively), whereas UFR contained 3.17% CBD. These values are generally consistent with the range previously reported for Futura 75 EOs (5.7–10.5%) [15,18], although substantially lower CBD levels (0.17%) have also been reported [21]. Differences among studies may partly reflect methodological factors, as the CBD content of distilled oils has been shown to vary with distillation time [18]. Trace GC peaks were tentatively assigned to Δ8-tetrahydrocannabinol (0.01% in UFL and LFL) and Δ9-tetrahydrocannabinol (0.01% in UFL and 0.05% in LFL). These assignments should be interpreted cautiously because analyte-specific authentic standards were not used. Owing to their low volatility, cannabinoids are only partially transferred to hemp EOs during hydrodistillation, while heating may promote decarboxylation of acidic cannabinoid precursors to neutral forms; high-temperature GC analysis may additionally affect the apparent acidic-to-neutral cannabinoid profile [18,47,48]. Consequently, these trace signals cannot be regarded as definitive evidence of the native occurrence and abundance of Δ8-THC and Δ9-THC in the original plant material. Confirmation would require targeted LC-based analysis using authentic standards.

### 2.2. Cytotoxic Activity of Essential Oils

**Figure 1:**
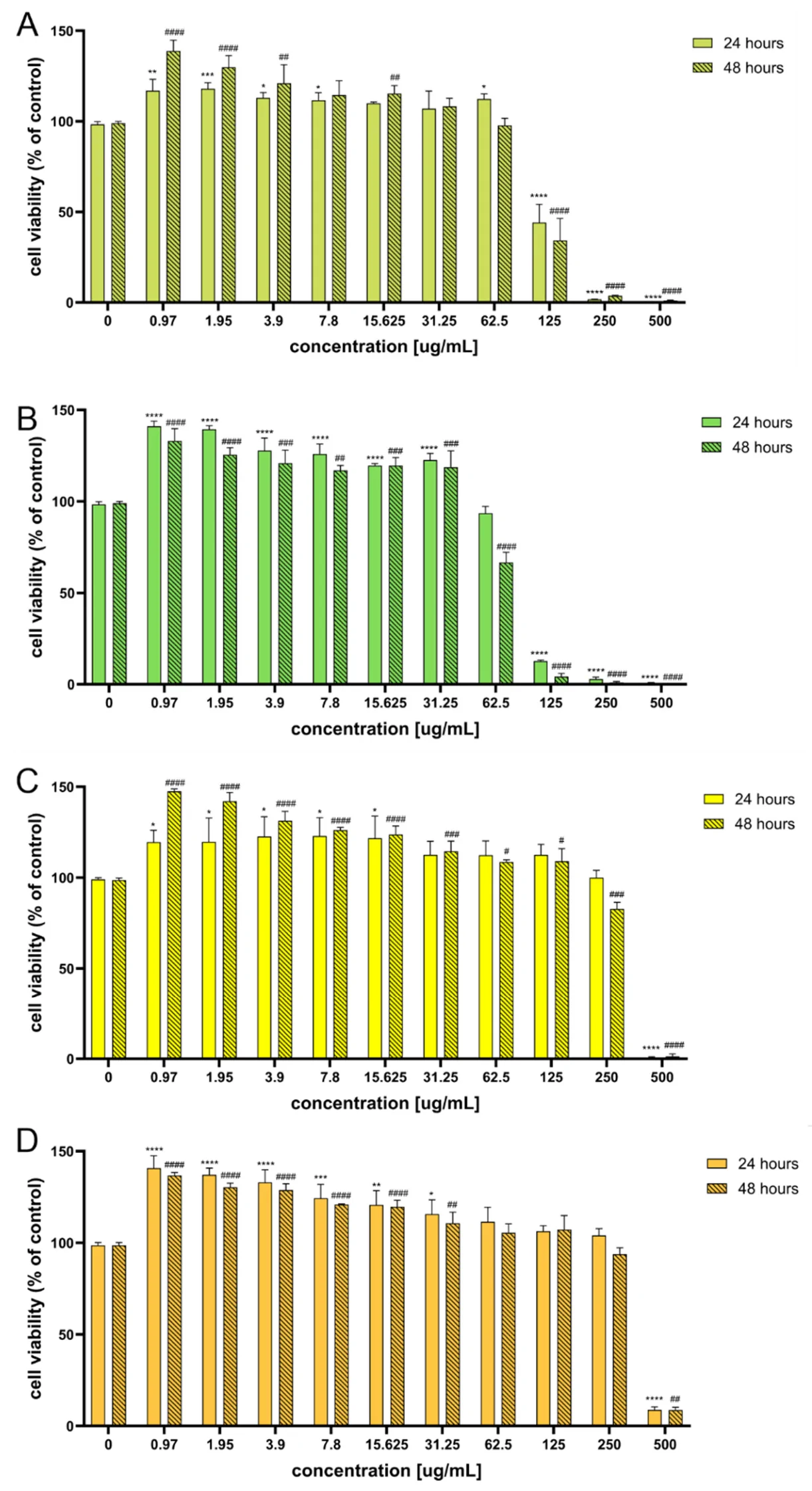
Cytotoxicity of hemp essential oils toward BJ human skin fibroblasts after 24 and 48 h of incubation with essential oils at concentrations ranging from 0.97 to 500 µg mL^−1^. Cell viability was determined using the MTT assay and expressed as the mean ± SD (*n* = 3). (**A**)—essential oils obtained from flowers, bracts and leaves of the upper part of the inflorescence; (**B**)—essential oils obtained from flowers, bracts and leaves from the lower part of the inflorescence; (**C**)—essential oils obtained from bracts and leaves from the upper part of the infructescence; (**D**)—essential oils obtained from bracts and leaves of the lower part of the infructescence. Statistical analysis was performed using one-way ANOVA followed by Dunnett’s multiple comparisons test. Statistical significance was assessed relative to the untreated control (cells cultured without the tested compound). For the 24 h incubation: *p* < 0.05 (*), *p* < 0.01 (**), *p* < 0.001 (***), and *p* < 0.0001 (****). For the 48 h incubation: *p* < 0.05 (^#^), *p* < 0.01 (^##^), *p* < 0.001 (^###^), and *p* < 0.0001 (^####^).

The effects of the EOs on normal human skin fibroblasts (BJ cell line) were first evaluated using the 3-(4,5-dimethylthiazol-2-yl)-2,5-diphenyltetrazolium bromide (MTT) assay. All tested EOs showed concentration-dependent effects on cellular metabolic activity (Figure 1).

Inflorescence-derived EOs (UFL and LFL) exerted the strongest inhibitory effects. A statistically significant reduction in the MTT signal was observed at concentrations ranging from 125 to 500 µg mL^−1^, with the strongest effect noted at 500 µg mL^−1^ after 48 h of exposure (UFL: 1.03%; LFL: 0% relative to the untreated control). For LFL, a significant reduction was also detected at 62.5 µg mL^−1^ after 48 h (66.6% of the control). In contrast, infructescence-derived EOs (UFR and LFR) displayed considerably weaker effects, with a significant reduction in the MTT signal observed only at the highest tested concentration (500 µg mL^−1^). At this concentration, the MTT signal decreased to 1.3% ± 1.4 for UFR and 8.7% ± 1.5 for LFR after 48 h of exposure. Overall, these results indicate that inflorescence-derived EOs exhibit greater cytotoxic potential than infructescence-derived EOs under the applied experimental conditions, with the response depending on both concentration and exposure time.

Interestingly, at lower EO concentrations (0.97–31.25 µg mL^−1^), the MTT signal exceeded 100% relative to the untreated control. This increase may reflect enhanced metabolic activity and/or a higher number of viable BJ fibroblasts; however, the MTT assay alone does not allow these possibilities to be distinguished and therefore provides no direct evidence of increased cell proliferation. Accordingly, the increased MTT signal observed at low EO concentrations should be interpreted cautiously and requires further investigation using direct measures of cell proliferation.

The concentration-dependent cytotoxicity observed in the present study is consistent with the general behaviour reported for many plant-derived EOs. Due to their complex chemical composition and high content of biologically active volatile compounds, EOs often exert dose-dependent effects on mammalian cells, where low concentrations may be well tolerated or produce an increased metabolic signal, whereas higher concentrations reduce cell viability. Similar dose-dependent cytotoxic or antiproliferative effects have been reported for EOs obtained from various plant species, including *Thymus vulgaris* [49], *Satureja intermedia* [50], and *Callistemon citrinus* [51]. Similarly, hemp EOs have shown dose-dependent effects on other cell models, including cholangiocytes and cancer cell lines [23]. Although these studies were not performed using fibroblasts, they illustrate the strong dependence of EO effects on concentration and cellular model. Direct evidence regarding hemp EOs and normal human skin fibroblasts remains limited; however, Aguzzi et al. [52] demonstrated that the cytotoxicological profile of hemp EOs and their nanoemulsions in cell models relevant to topical and inhalation exposure depended on concentration, formulation, and cell type. Studies using other hemp-derived preparations provide additional context: hemp herb extracts have shown low cytotoxicity toward skin cells, including fibroblasts [53], while hemp extract and cannabidiol have been reported to modulate inflammatory mediators in human keratinocytes and fibroblasts [54] and to promote wound-healing activity in human gingival fibroblasts [55]. Although these findings cannot be directly extrapolated to the EOs investigated here, they support further investigation of the biological effects of hemp-derived preparations in fibroblast models. In the present study, the markedly lower cytotoxicity of UFR and LFR toward BJ fibroblasts compared with inflorescence-derived EOs indicates greater cytocompatibility of infructescence-derived EOs under the applied experimental conditions and supports their further evaluation for potential topical applications.

**Figure 2:**
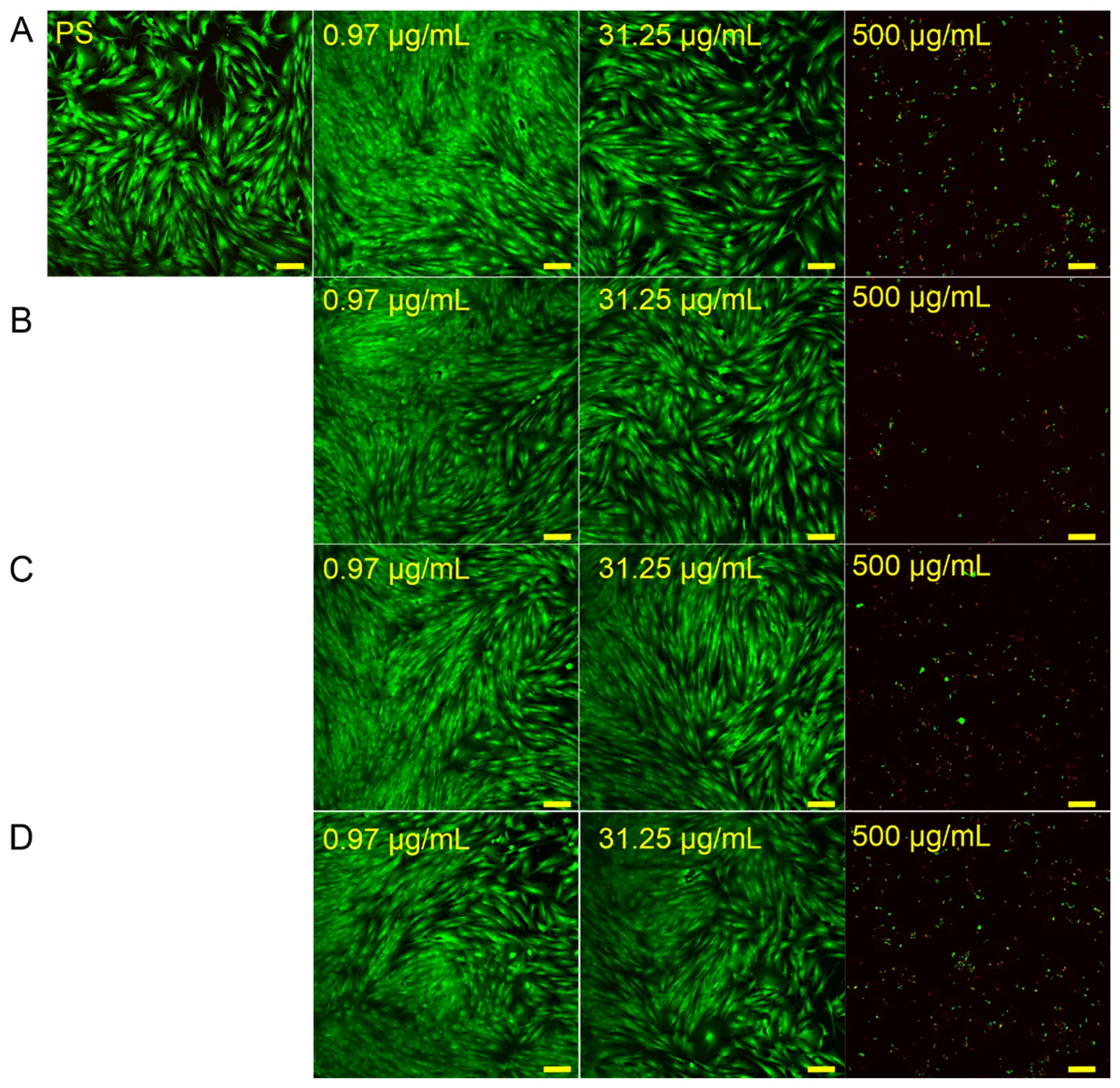
Representative confocal laser scanning microscopy (CLSM) images of Live/Dead staining of BJ human skin fibroblasts after 24 h of incubation with the tested compounds at concentrations of 0.97, 31.25, and 500 µg mL^−1^. (**A**)—essential oils obtained from flowers, bracts and leaves of the upper part of the inflorescence; (**B**)—essential oils obtained from flowers, bracts and leaves from the lower part of the inflorescence; (**C**)—essential oils obtained from bracts and leaves from the upper part of the infructescence; (**D**)—essential oils obtained from bracts and leaves of the lower part of the infructescence. PS—negative control (cells cultured on tissue culture polystyrene, without EO treatment). Live cells are shown in green, whereas dead cells are shown in red. Images were acquired at 100× magnification. Scale bar = 100 µm.

Live/Dead fluorescence staining combined with confocal laser scanning microscopy (CLSM) provided complementary evidence of the concentration-dependent effects obtained in the MTT assay (Figure 2).

After 24 h of incubation, cells exposed to the lowest tested concentration (0.97 µg mL^−1^) appeared more densely distributed than untreated control cells. They retained typical elongated, spindle-shaped fibroblast morphology and showed predominantly green fluorescence, indicating high viability, with only occasional red-stained dead cells. A similar effect was observed at 31.25 µg mL^−1^, with preserved fibroblast morphology and predominantly green fluorescence, indicating no detectable cytotoxic effects at this concentration. These observations were consistent with the increased tetrazolium reduction observed at lower EO concentrations in the MTT assay. However, because the Live/Dead images were evaluated qualitatively without direct cell counting, the apparently higher cell density should not be interpreted as definitive evidence of increased cell proliferation. In contrast, exposure to the highest tested concentration (500 µg mL^−1^) resulted in pronounced cytotoxicity, characterised by numerous red-stained dead cells and only a limited number of viable green cells. Moreover, the remaining viable cells displayed marked morphological alterations, including loss of their characteristic fibroblast-like shape and adoption of a rounded morphology, indicating severe cellular damage.

The pronounced morphological alterations observed after exposure to the highest EO concentration may be associated with the cytotoxic effect of volatile terpenoid compounds present in the oils [56]. Owing to their lipophilic nature, EO constituents can interact with cellular membranes, potentially disturbing membrane integrity and intracellular homeostasis [57,58]. In eukaryotic cells, EOs may also act as pro-oxidants and affect intracellular membranes and organelles, including mitochondria [56,57]. At high concentrations, these effects may lead to cell shrinkage, rounding, loss of the typical fibroblast-like morphology, and increased membrane permeability, consistent with the presence of red-stained dead cells in the Live/Dead assay. Therefore, the CLSM observations indicate that the reduced metabolic activity detected by the MTT assay at 500 µg mL^−1^ was accompanied by loss of membrane integrity and pronounced morphological alterations in BJ fibroblasts.

**Figure 3:**
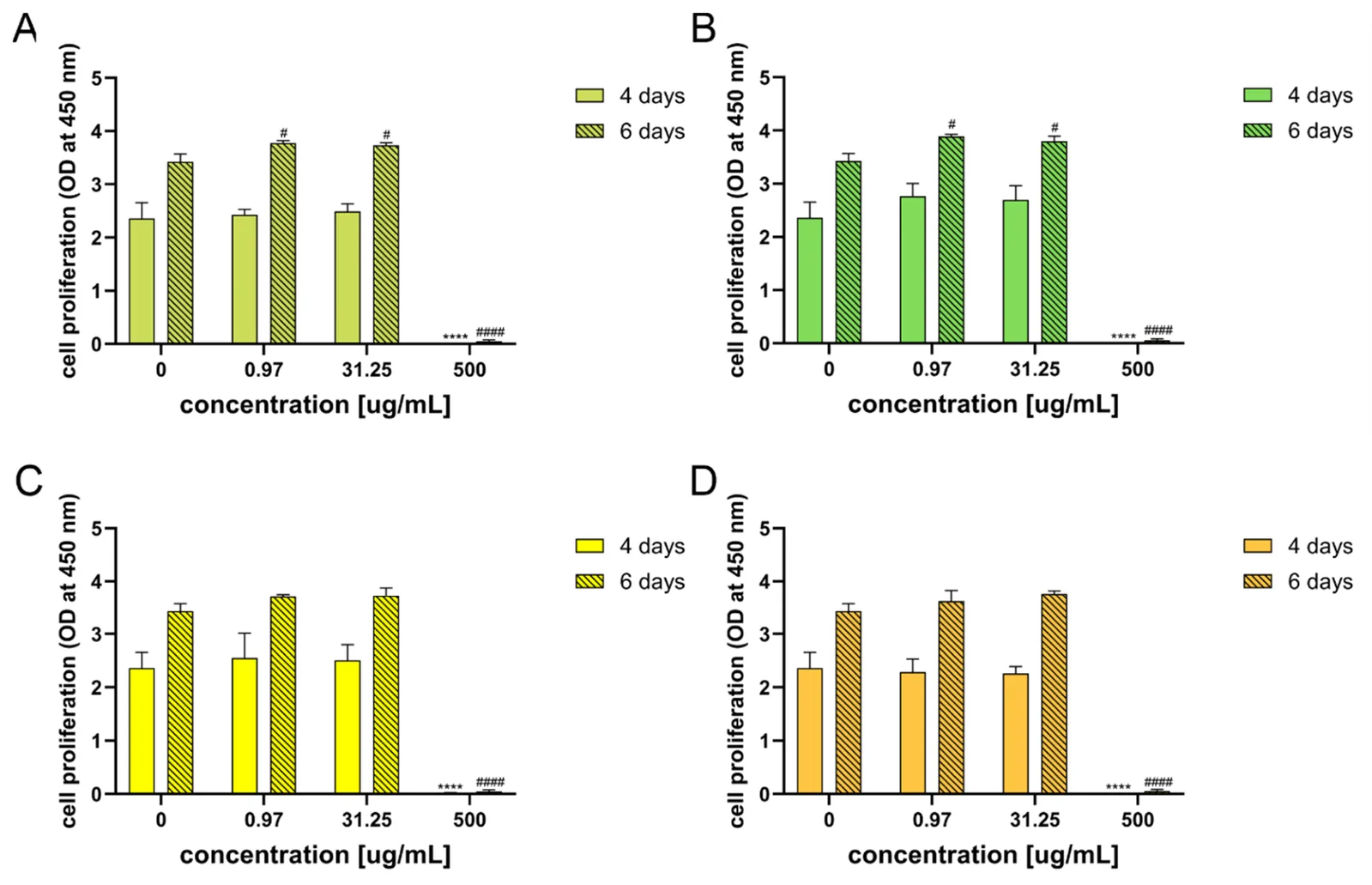
Effect of hemp essential oils on the proliferative activity of BJ human skin fibroblasts after 4 and 6 days of incubation, assessed using the water-soluble tetrazolium salt (WST-8) assay. Cell proliferation is presented as optical density at 450 nm (OD450) and expressed as the mean ± SD (*n* = 3). (**A**)—essential oils obtained from flowers, bracts and leaves of the upper part of the inflorescence; (**B**)—essential oils obtained from flowers, bracts and leaves from the lower part of the inflorescence; (**C**)—essential oils obtained from bracts and leaves from the upper part of the infructescence; (**D**)—essential oils obtained from bracts and leaves of the lower part of the infructescence. Statistical analysis was performed using one-way ANOVA followed by Dunnett’s multiple comparisons test. Statistical significance was assessed relative to the untreated control corresponding to the respective incubation time. For samples incubated for 4 days, statistical significance is indicated as *p* < 0.0001 (****). For samples incubated for 6 days, statistical significance is indicated as *p* < 0.05 (#) and *p* < 0.0001 (####).

The long-term effects of the EOs on BJ fibroblasts were further evaluated using the water-soluble tetrazolium salt (WST-8) assay after 4 and 6 days of incubation (Figure 3). At the low and intermediate concentrations (0.97 and 31.25 µg mL^−1^), the WST-8 signal after 4 days generally remained comparable to that of the untreated control, although a slight, non-significant increase was observed for LFL and UFR. After 6 days, a significant increase in the WST-8 signal was detected for UFL and LFL compared with the control. As with the MTT assay, this increased tetrazolium reduction may reflect enhanced metabolic activity and/or a higher number of viable cells but cannot be interpreted as direct evidence of increased proliferation. The increased WST-8 signal after prolonged exposure does not contradict the short-term MTT results, as both assays were performed under different experimental conditions, including different initial cell densities and incubation times. Thus, the MTT and WST-8 assays provide complementary information on the short- and longer-term cellular responses, respectively, rather than directly comparable measures. In contrast, the highest tested concentration (500 µg mL^−1^) caused a marked and persistent reduction in the WST-8 signal after both 4 and 6 days, indicating that the inhibitory effect observed at this concentration persisted throughout the experiment.

Previous studies provide a broader context for investigating the effects of hemp-derived preparations and plant EOs. Kongkadee et al. [55] showed that hemp extract and CBD promoted wound healing activity in human gingival fibroblasts, while CBD-based biomaterials have shown potential to support wound healing processes in vitro [59]. Similarly, lavender EO has been associated with increased collagen synthesis and fibroblast differentiation during wound healing, and EO from *Eugenia dysenterica* leaves exhibited wound-healing activity in L929 fibroblasts [60,61]. However, these findings cannot be directly extrapolated to the EOs investigated in the present study. The increased WST-8 signal observed at low EO concentrations provides no direct evidence of regenerative or wound-healing activity, and further studies using specific functional assays, such as cell proliferation, migration, and wound-closure assays, would be required to evaluate such effects.

**Figure 4:**
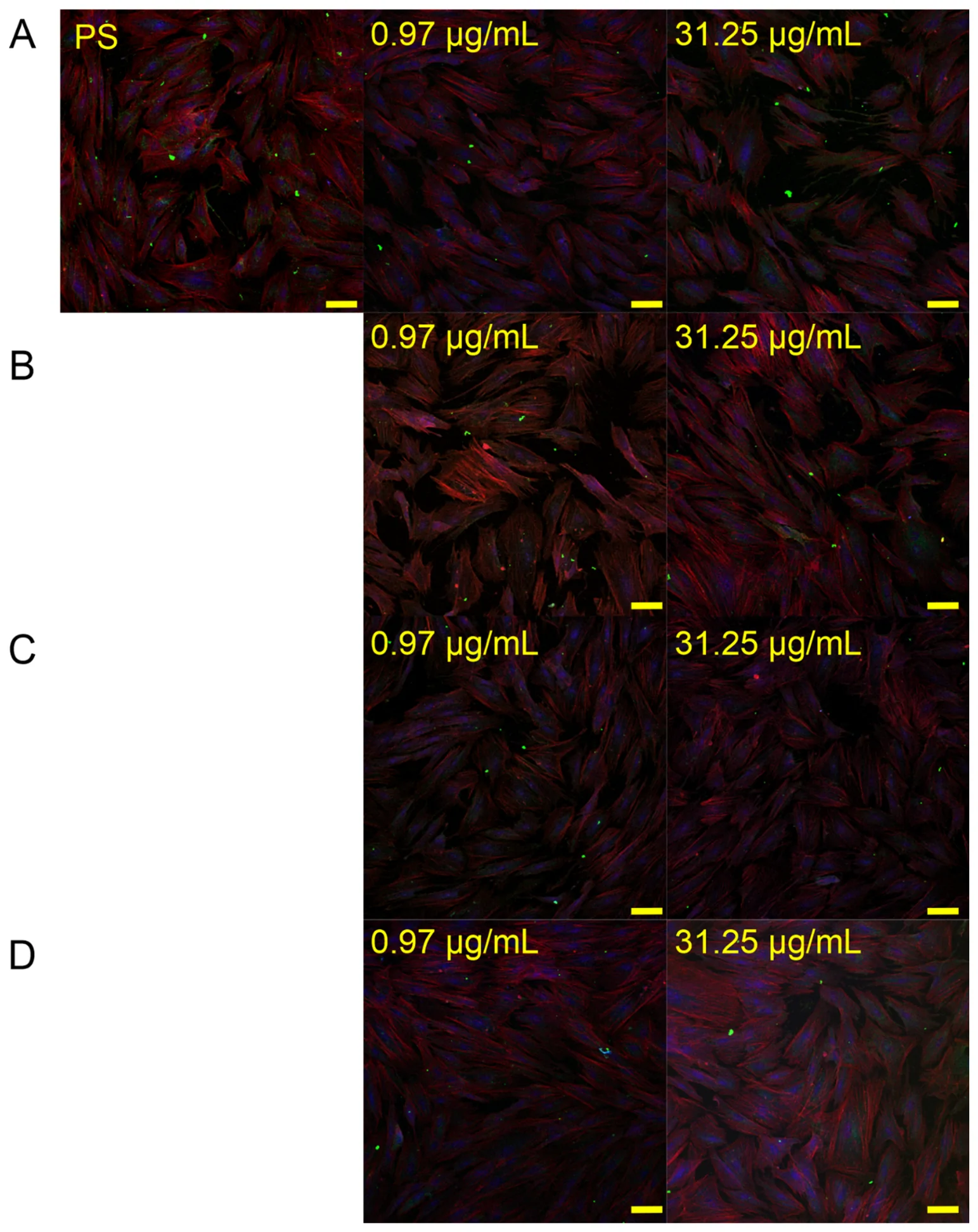
Representative confocal laser scanning microscopy (CLSM) images of immunofluorescence staining of BJ human skin fibroblasts after 4 days of incubation with essential oils obtained from UFL, LFL, UFR and LFR at concentrations of 0.97 and 31.25 µg mL^−1^. Cell nuclei were stained with Hoechst 33342 (blue fluorescence), the cytoskeleton/F-actin was stained with Alexa Fluor 635 (red fluorescence), and collagen type I was visualized using an Alexa Fluor 488-conjugated secondary antibody (green fluorescence). Images were acquired at 200× magnification. Scale bar = 100 µm. (**A**)—essential oils obtained from flowers, bracts and leaves of the upper part of the inflorescence; (**B**)—essential oils obtained from flowers, bracts and leaves from the lower part of the inflorescence; (**C**)—essential oils obtained from bracts and leaves from the upper part of the infructescence; (**D**)—essential oils obtained from bracts and leaves of the lower part of the infructescence. PS—negative control (cells cultured on tissue culture polystyrene, without EO treatment).

To qualitatively evaluate the effects of the EOs on extracellular matrix (ECM)-related staining and cellular morphology, BJ fibroblasts were stained for F-actin, type I collagen, and cell nuclei and subsequently analysed by CLSM (Figure 4). The analysis was performed in three independent experiments (*n* = 3), with multiple microscopic fields examined for each experimental condition. Representative images reflecting the staining pattern consistently observed across the independent experiments were selected for presentation. All images within a given assay were acquired using the same microscope and image-processing settings.

The qualitative analysis indicated that treatment with the selected EO concentrations did not induce detectable alterations in cell morphology, cytoskeletal organization, or type I collagen distribution. The cells retained their characteristic elongated, spindle-shaped fibroblast-like morphology, with a well-organized actin cytoskeleton and uniformly distributed nuclei. Moreover, the intensity and distribution of type I collagen staining were comparable to those observed in the untreated control, indicating no visible changes under the tested conditions. BJ fibroblasts exposed to the highest concentration of 500 µg mL^−1^ were also subjected to fluorescence staining. However, the pronounced cytotoxic effect and insufficient number of adherent cells remaining after 4 days of incubation precluded the acquisition of representative CLSM images. These observations are consistent with the marked reduction in cellular metabolic activity detected at the highest tested concentration in the WST-8 assay. Preservation of fibroblast morphology, cytoskeletal organization, and type I collagen distribution at the selected EO concentrations provides additional evidence of their cytocompatibility under the applied experimental conditions. Fibroblast morphology and actin cytoskeletal integrity are closely related to fundamental cellular processes such as adhesion and migration, while type I collagen is a major component of the extracellular matrix [54,55,60]. However, the qualitative preservation of these structural features should not be interpreted as evidence of increased collagen synthesis, enhanced fibroblast function, or wound-healing activity. Rather, the present observations indicate that the tested EO concentrations did not induce detectable alterations in the examined structural features of BJ fibroblasts. Together with the metabolic activity and Live/Dead results, these findings further support the greater cytocompatibility of infructescence-derived EOs and provide a basis for their further investigation in skin-related in vitro models using direct functional assays.

### 2.3. Haemolytic Activity of the Tested Essential Oils

**Figure 5:**
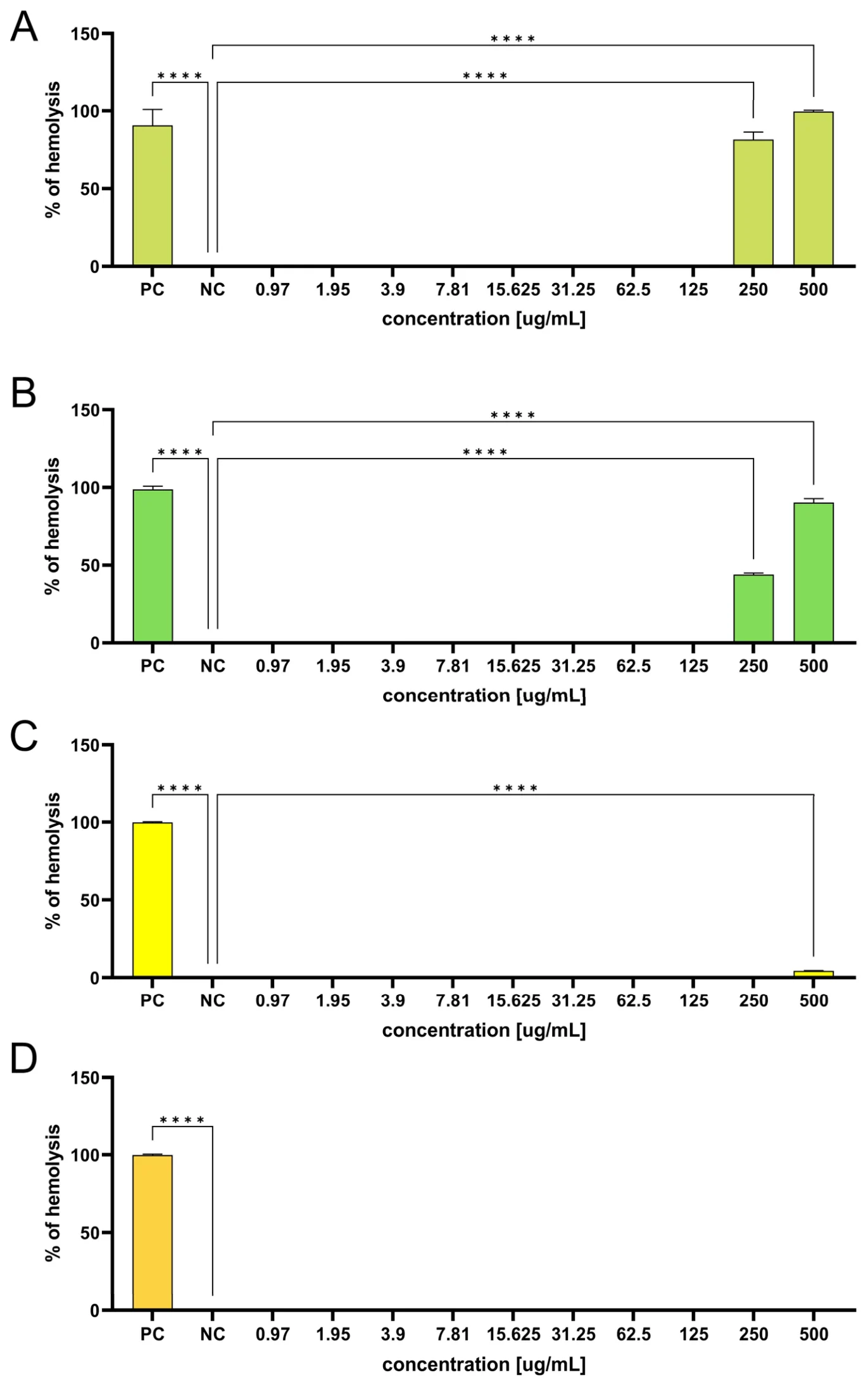
Haemolytic activity of hemp essential oils at concentrations ranging from 0.97 to 500 µg mL^−1^. Haemolysis is expressed as a percentage relative to the positive control (PC; blood treated with 0.1% Triton X-100), defined as 100% haemolysis, while the negative control (NC; blood without the tested compound) represented 0% haemolysis. Data are presented as the mean ± SD (*n* = 4). (**A**)—essential oils obtained from flowers, bracts and leaves of the upper part of the inflorescence; (**B**)—essential oils obtained from flowers, bracts and leaves from the lower part of the inflorescence; (**C**)—essential oils obtained from bracts and leaves from the upper part of the infructescence; (**D**)—essential oils obtained from bracts and leaves of the lower part of the infructescence. Statistical analysis was performed using one-way ANOVA followed by Dunnett’s multiple comparisons test. Statistical significance was assessed relative to the negative control (NC): *p* < 0.0001 (****).

The haemolytic activity of the tested EOs was evaluated over a concentration range of 0.97–500 µg mL^−1^. For comparative interpretation, the ASTM F756 thresholds were applied, according to which haemolysis of <2%, 2–5%, and >5% corresponds to non-haemolytic, slightly haemolytic, and haemolytic ranges, respectively [62]. A clear concentration-dependent effect on erythrocyte membrane integrity was observed for all tested samples (Figure 5). Inflorescence-derived EOs (UFL and LFL) exhibited pronounced haemolytic activity at higher concentrations.

At 250 µg mL^−1^, LFL and UFL induced 43.92% and 81.51% haemolysis, respectively, which increased to 90.27% and 99.54% at 500 µg mL^−1^ (*p* < 0.0001 vs. the negative control). According to the ASTM F756 classification thresholds applied as a comparative framework, both EOs were classified as haemolytic at these concentrations. In contrast, infructescence-derived EOs (UFR and LFR) demonstrated markedly improved hemocompatibility. Essential oil obtained from LFR did not induce detectable haemolysis at any of the tested concentrations, whereas EO obtained from UFR caused only 4.49% haemolysis at 500 µg mL^−1^ and no relevant increase at lower concentrations. Thus, according to the same comparative thresholds, LFR remained within the non-haemolytic range throughout the tested concentration range, while UFR was within the slightly haemolytic range only at 500 µg mL^−1^.

Hemocompatibility is an important safety parameter when considering potential biomedical or topical applications of EOs. The haemolytic activity observed at high concentrations, particularly for UFR and LFR, is consistent with previous reports showing that EOs and their terpene constituents can interact with erythrocyte membranes. Due to their lipophilic character, EO components may incorporate into lipid bilayers, increase membrane fluidity, disturb membrane integrity, and ultimately promote erythrocyte lysis [63,64,65]. Therefore, the strong haemolysis induced by UFL and LFL EOs at high concentrations may reflect a pronounced interaction of their volatile constituents with red blood cell membranes. In contrast, the markedly lower haemolytic activity of UFR and LFR suggests a less disruptive interaction under the tested conditions. Cannabis-derived compounds have also been reported to affect blood cells in a concentration-dependent manner: a cannabinoid mixture increased free haemoglobin levels and affected platelet-related parameters in vitro [66], while CBD was shown to alter erythrocyte integrity and induce haemolytic vesicle formation [67]. Against this background, the negligible haemolytic activity of EO obtained from LFR and the minimal haemolysis induced by EO obtained from UFR indicate greater hemocompatibility of the infructescence-derived EOs compared with UFL and LFL under the applied experimental conditions. However, as blood was obtained from a single healthy volunteer, interindividual variability in erythrocyte susceptibility could not be assessed, and these findings should be considered preliminary.

**Figure 6:**
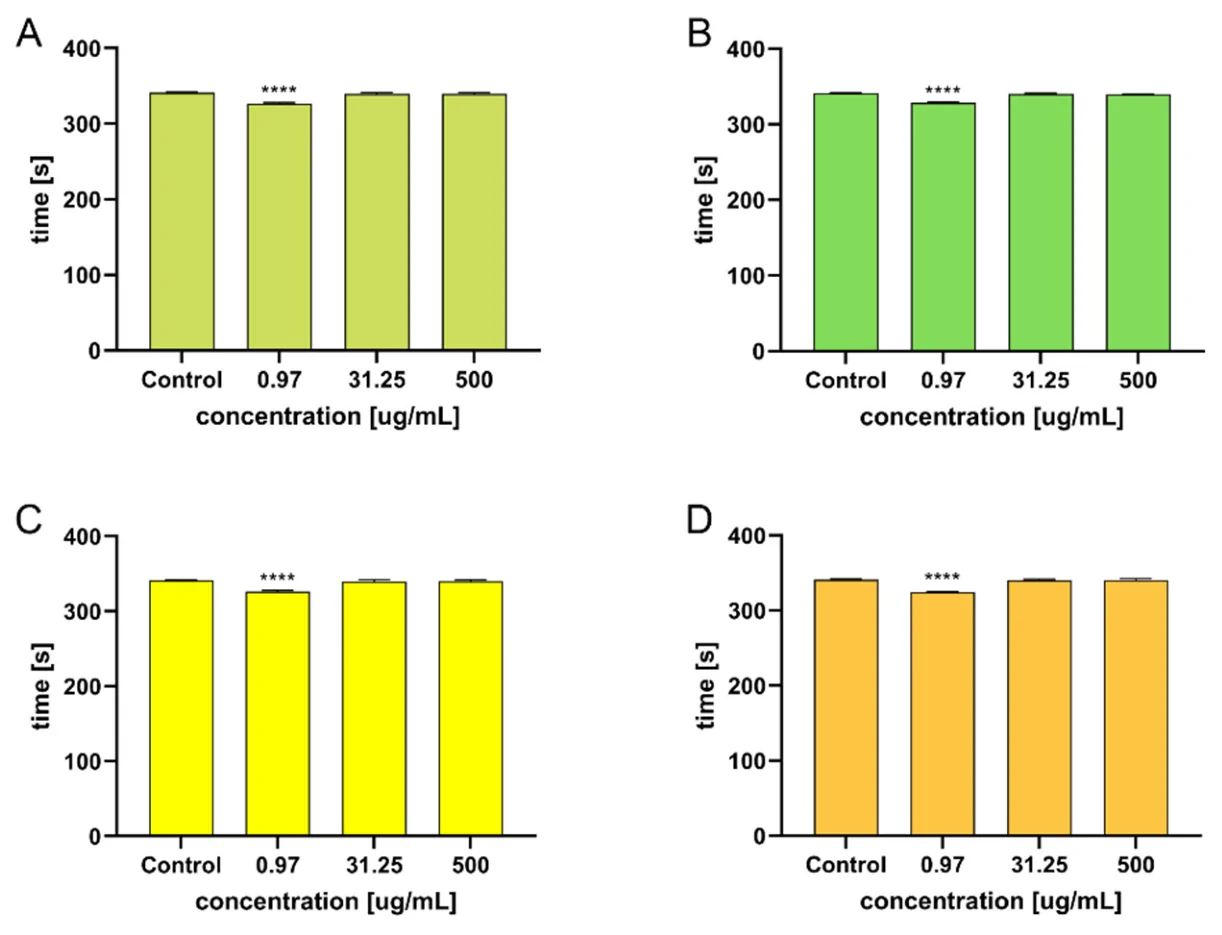
Effect of hemp essential oil compounds on blood clotting time. Blood clotting time is expressed in seconds and presented as the mean ± SD (*n* = 3). The control represents blood activated with CaCl_2_ in the absence of the tested compounds. (**A**)—essential oils obtained from flowers, bracts and leaves of the upper part of the inflorescence; (**B**)—essential oils obtained from flowers, bracts and leaves from the lower part of the inflorescence; (**C**)—essential oils obtained from bracts and leaves from the upper part of the infructescence; (**D**)—essential oils obtained from bracts and leaves of the lower part of the infructescence. Statistical analysis was performed using one-way ANOVA followed by Dunnett’s multiple comparisons test. Statistical significance was assessed relative to the control: *p* < 0.0001 (****).

The effect of the tested EOs on blood coagulation was evaluated by measuring clot formation time. At the lowest tested concentration (0.97 µg mL^−1^), all four EOs significantly shortened the manually determined clot formation time by approximately 10 s compared with the control(*p* < 0.0001) (Figure 6). Considering the limited magnitude of this difference and the operator-dependent nature of the assay, this observation should be regarded as a preliminary indication of a possible effect on coagulation rather than conclusive evidence of procoagulant activity. In contrast, no statistically significant differences in clotting time were observed at the remaining tested concentrations (*p* > 0.05). Further studies using standardized automated coagulation assays would be needed to confirm the biological relevance and reproducibility of the observed difference.

Previous studies concerning hemp-derived compounds have mostly reported anticoagulant or antiplatelet rather than procoagulant effects. For example, hemp extracts and selected cannabinoids have been shown to prolong clotting time in experimental models, while CBD and CBG reduced platelet aggregation and thrombus formation under flow conditions [68,69]. Similarly, several EOs and their terpene constituents have been described as modulators of platelet activation and coagulation pathways, often showing anticoagulant or antiplatelet activity [70,71]. In this context, the absence of prolonged clot formation time in the present study suggests that the tested hemp EOs did not impair coagulation under the investigated conditions. The modest shortening of clotting time observed only at 0.97 µg mL^−1^ should be interpreted cautiously, particularly given the lack of a concentration-dependent response. Its biological relevance would need to be evaluated using standardized coagulation assays, such as prothrombin time (PT) and activated partial thromboplastin time (aPTT), together with more specific analyses of platelet function.

### 2.4. Antibacterial Activity of Essential Oils

**Figure 7:**
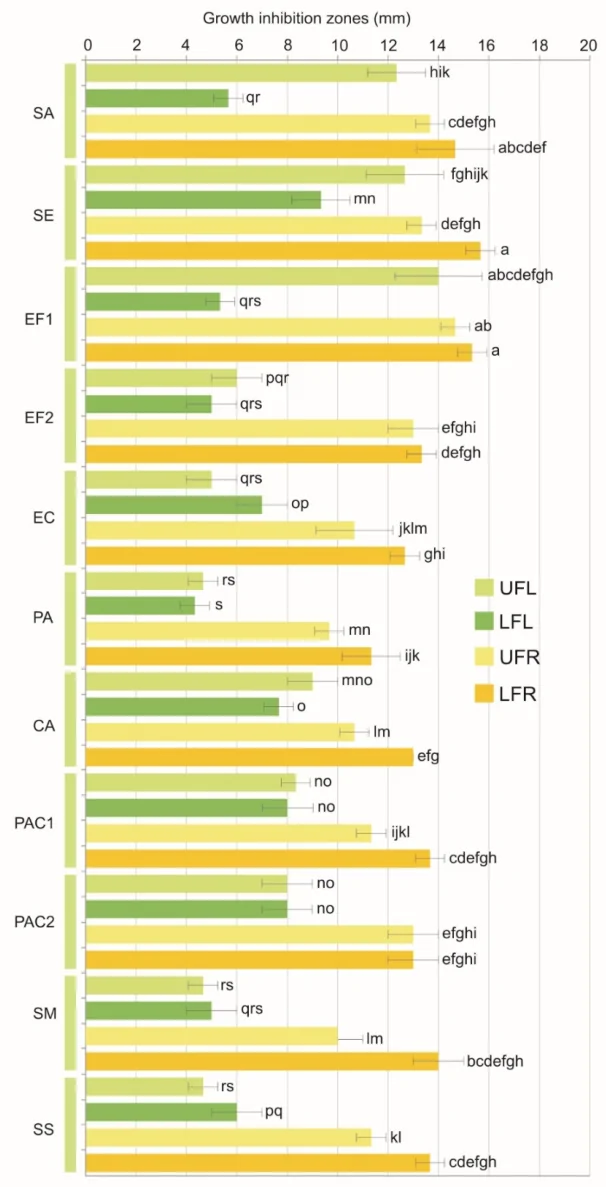
Zones of bacterial growth inhibition induced by the hemp essential oils. SA—*S. aureus* ATCC 25923, SE—*S. epidermidis* ATCC 12228, EF1—*E. faecalis* PCM 896, EF2—*E. faecalis* (w.c.i.), EC—*E. coli* ATCC 25922, PA—*P. aeruginosa* ATCC 27853, CA—*C. acnes* ATCC 11827, PAC1—*C. acnes* PCM 2334, PAC2—*C. acnes* PCM 2400, SM—*S. mutans* PCM 2020, SS—*S. sanguinis* PCM 2335, UFL—flowers, bracts and leaves from the upper part of the inflorescence, LFL—flowers, bracts and leaves from the lower part of the inflorescence, UFR—bracts and leaves from the upper part of the infructescence, LFR—bracts and leaves from the lower part of the infructescence. The values designated by different letters are significantly different; two-way ANOVA followed by Tukey’s test, *p* < 0.05.

The agar diffusion assay revealed differences in growth inhibition among the tested bacterial strains and EO types (Figure 7). Two-way ANOVA showed a significant effect of EO type (F = 441.6, *p* < 0.001), bacterial strain (F = 35.3, *p* < 0.001), and their interaction (F = 9.4, *p* < 0.001) on inhibition zone diameter. Because the diffusion of EO constituents through agar depends on their physicochemical properties, inhibition zone diameters should be regarded as preliminary indicators of antibacterial activity rather than direct measures of antibacterial potency.

The antimicrobial activity of the tested hemp EOs varied markedly with EO type and bacterial strain. The highest activity was observed for infructescence-derived EOs, particularly LFR, which inhibited the growth of most tested strains at concentrations ranging from 0.025 to 0.20 mg mL^−1^ (Table 3). In contrast, inflorescence-derived EOs generally exhibited higher MIC values, with no inhibition detected within the tested concentration range for several strain–EO combinations.

Gram-positive bacteria were generally the most susceptible to the infructescence-derived EOs, especially *Staphylococcus epidermidis* (MIC 0.025–0.050 mg mL^−1^), *Staphylococcus aureus* (0.05–0.10 mg mL^−1^), and acne-associated bacteria (0.025–0.10 mg mL^−1^). LFR and UFR were also active against the oral streptococci *Streptococcus mutans* and *Streptococcus sanguinis*, with MIC values of 0.05–0.10 mg mL^−1^, whereas the corresponding MICs for inflorescence-derived EOs were up to 16–32-fold higher or exceeded the highest tested concentration. Although Gram-negative bacteria were generally less susceptible to the tested EOs, those obtained from UFR and LFR inhibited both *E. coli* and *P. aeruginosa* (MIC 0.20–0.40 mg mL^−1^), indicating a relatively broad spectrum of antimicrobial activity. It should be highlighted that, for several strains, the MIC values of the inflorescence-derived EOs exceeded the highest tested concentration (1.60 mg mL^−1^), suggesting that the observed superiority of the infructescence-derived EOs may have been underestimated. It is also noteworthy that antimicrobial efficacy appeared to be more strongly influenced by the type of EO than by the taxonomic affiliation of the bacterial strains, with UFR and particularly LFR showing greater activity against most tested strains. Sparfloxacin, used as the reference antimicrobial agent, exhibited markedly lower MIC values than the tested EOs, confirming the validity of the assay.

**Figure 8:**
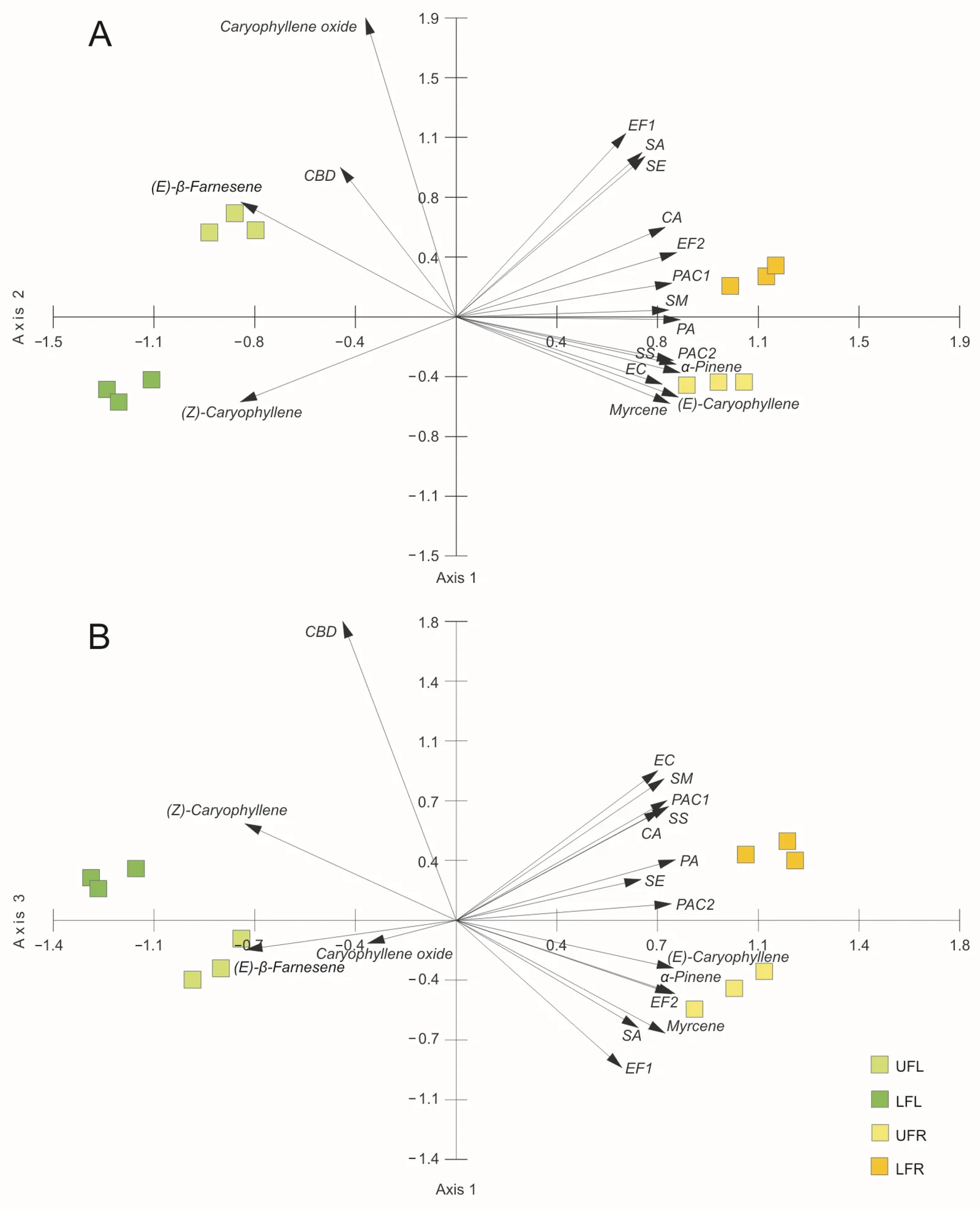
PCA ordination on the basis of the chemical composition of *C. sativa* essential oils and zones of bacterial growth inhibition induced by the mentioned EO. (**A**)—ordination space of Axis 1 and Axis 2, (**B**)—ordination space of Axis 1 and Axis 3. SA—*S. aureus* ATCC 25923, SE—*S. epidermidis* ATCC 12228, EF1—*E. faecalis* PCM 896, EF2—*E. faecalis* (w.c.i.), EC—*E. coli* ATCC 25922, PA—*P. aeruginosa* ATCC 27853, CA—*C. acnes* ATCC 11827, PAC1—*C. acnes* PCM 2334, PAC2—*C. acnes* PCM 2400, SM—*S. mutans* PCM 2020, SS—*S. sanguinis* PCM 2335, UFL—flowers, bracts and leaves from the upper part of the inflorescence, LFL—flowers, bracts and leaves from the lower part of the inflorescence, UFR—bracts and leaves from the upper part of the infructescence, LFR—bracts and leaves from the lower part of the infructescence.

Principal component analysis (PCA) was used to explore associations between the chemical composition of the hemp EOs and bacterial growth inhibition zones (Figure 8). The first three axes (PCs) explained 97.42% of the total variance (76.03% for Axis 1, 13.41% for Axis 2, and 7.98% for Axis 3) (Table 4). PC1 primarily differentiated EOs associated with higher contents of α-Pinene, Myrcene, and (E)-Caryophyllene from those associated with higher contents of (Z)-Caryophyllene and (E)-β-Farnesene. Growth inhibition zones for all tested bacterial strains loaded positively on PC1, indicating that greater antibacterial activity was associated with the former compositional profile. This association was particularly strong for PA, EF2, PAC2, and SS, whereas EF1, SA, and SE showed relatively greater contributions to Axis 2. PC2 was mainly associated with Caryophyllene oxide and differentiated the responses of SA, SE, EF1, and CA from those of EC, PAC2, and SS. PC3 was primarily associated with CBD and further differentiated the responses of EC, SM, PAC1, and SS from those of EF1 and SA. Overall, the PCA revealed a clear association between EO composition and antibacterial activity: the broader activity of UFR and particularly LFR was associated with higher proportions of α-Pinene, Myrcene, and (E)-Caryophyllene, whereas the more selective activity of UFL was associated with a distinct compositional profile, including higher proportions of (Z)-Caryophyllene and Caryophyllene oxide. These multivariate associations should not, however, be interpreted as evidence of a causal contribution of individual EO constituents to antibacterial activity.

Previous studies have demonstrated antimicrobial activity of hemp EOs, although the investigated microorganisms and EO profiles varied considerably among studies. Zengin et al. [23] reported activity of EOs from Futura 75 against *S. aureus* ATCC 29213 and clinical *S. aureus* isolates, including effects assessed by MIC, MBC, and antibiofilm analyses. A broader screening of fibre-type hemp EOs, including Futura 75, demonstrated activity against several Gram-positive bacteria, including *S. aureus*, *S. epidermidis*, *Enterococcus faecalis*, *E. faecium*, *Listeria* spp. and *Bacillus* spp. [12]. Similarly, comparative analyses of Carmagnola, Fibranova, and Futura EOs against Gram-positive and Gram-negative bacteria and yeasts identified Futura as the most active of the three cultivars [11]. These studies provide a useful context for the present results while also highlighting the substantial influence of cultivar, EO composition, and bacterial strain on the observed antibacterial activity.

The present study extends the available evidence on the antibacterial activity of Futura 75 EOs to bacterial strains that have received limited attention in previous studies. The strongest activity was observed for EO obtained from LFR, which produced the highest inhibition zones for almost all tested strains, including *S. aureus* ATCC 25923, *S. epidermidis* ATCC 12228 and *E. faecalis* PCM 896 (14.67, 15.67, and 15.43 mm, respectively). Although *Staphylococcus* spp. and *Enterococcus* spp. have previously been included in studies on hemp EOs’ antibacterial activity, strain-specific data for Futura 75 remain limited. Šovljanski et al. [22] tested *S. aureus* ATCC 25923 and *P. aeruginosa* ATCC 27853 using EO from industrial hemp cv. Helena. Therefore, the present results extend the strain-specific evidence available for the antibacterial activity of Futura 75 EOs.

The anti-staphylococcal activity observed in the present study is consistent with previous reports, although its magnitude varied markedly among the tested EOs. Zengin et al. [23] reported MIC and MBC values of 8 and 16 mg mL^−1^, respectively, for Futura 75 EOs against *S. aureus*, whereas Iseppi et al. [12] found substantial variation in anti-staphylococcal activity among EOs from different hemp cultivars. In the present study, UFR and particularly LFR showed considerably lower MIC values against *S. aureus* and *S. epidermidis* than UFL and LFL. Although direct quantitative comparisons among studies should be made cautiously because of differences in EO composition and experimental conditions, these findings further indicate that anti-staphylococcal activity is strongly influenced by the chemical profile of the EO.

The results obtained for *E. faecalis* PCM 896 further support the sensitivity of enterococci to hemp EOs. Antibacterial activity against *Enterococcus hirae*, *E. faecium*, and *E. faecalis* has been reported for EOs from different hemp cultivars [11,12], while Šovljanski et al. [22] identified *E. faecalis* as one of the most susceptible bacteria to industrial hemp EO. In the present study, EOs obtained from LFR, UFR and UFL exhibited high activity against *E. faecalis* PCM 896, whereas the EO obtained from LFL was considerably less active. These findings support the susceptibility of selected *Enterococcus* strains to hemp EOs, while further indicating that the magnitude of the antibacterial efficacy depends strongly on EO composition.

A particularly important finding of the present study is the activity of hemp EOs against Gram-negative bacteria. Earlier studies often reported lower susceptibility of Gram-negative bacteria to hemp EOs, which is commonly attributed to the outer membrane barrier. Consistent with this general pattern, Šovljanski et al. [22] found no activity of EO from industrial hemp cv. Helena against Gram-negative bacteria, yeasts or fungi. In contrast, Zheljazkov et al. [72] reported strong antimicrobial activity of selected hemp chemovar EOs against several Gram-negative and foodborne bacteria (including *E. coli*, *Salmonella enterica* and *Listeria monocytogenes*), whereas Nafis et al. [73] demonstrated inhibition of *E. coli* and *P. aeruginosa* by Moroccan hemp EO, with MIC values of 1.2 mg mL^−1^ for both strains. In this context, the activity of Futura 75 EOs obtained from LFR and UFR against *E. coli* ATCC 25922 and *P. aeruginosa* ATCC 27853 is particularly noteworthy. The activity against *P. aeruginosa* ATCC 27853 is especially interesting because the same strain was resistant to the hemp EO tested by Šovljanski et al. [22]. Together, these contrasting findings indicate that the susceptibility of Gram-negative bacteria to hemp EOs may vary substantially depending on EO composition and other experimental factors.

Relatively few studies have investigated the activity of industrial hemp EOs against acne-associated bacteria [11,12,18,21,22,23,47,72,73,74]. In the present study, EOs obtained from LFR showed pronounced activity against the tested acne-associated strains, producing inhibition zones of 13.00 mm against *C. acnes* ATCC 11827 and 13.67 mm against *C. acnes* PCM 2334. EOs obtained from both LFR and UFR also inhibited *C. acnes* PCM 2400, with inhibition zones of 13.00 mm. These findings extend the available evidence on the antibacterial spectrum of Futura 75 EOs to acne-associated bacterial strains. However, their relevance to potential dermatological applications requires further investigation using complementary approaches, including bactericidal, time-kill, and antibiofilm assays, as well as appropriate skin-related experimental models.

The activity of the tested hemp EOs against oral streptococci represents another relevant finding of the present study. Essential oil obtained from LFR inhibited *S. mutans* PCM 2020 and *S. sanguinis* PCM 2335, producing inhibition zones of 14.00 and 13.67 mm, respectively. Nissen et al. [11] previously evaluated hemp EOs against another streptococcal species, *Streptococcus salivarius* subsp. *thermophilus*, whereas evidence concerning the activity of Futura 75 EOs against oral streptococci remains limited. The present findings therefore extend the antibacterial spectrum demonstrated for Futura 75 EOs to the tested oral streptococci and provide a rationale for further studies addressing cariogenic biofilms, bacterial adhesion, and interactions within multispecies oral microbiota.

The variability observed among the studied EOs is consistent with previous reports showing substantial cultivar- and composition-dependent differences in the antibacterial activity of hemp EOs. Novak et al. [74] showed considerable variation in EO composition among hemp cultivars accompanied by generally modest and cultivar-dependent antimicrobial activity. Similarly, substantial differences in the proportions of major sesquiterpenes have been reported among novel Serbian hemp chemovars [72], while Palmieri et al. [18] demonstrated that both cultivar and distillation time may influence EO composition and antimicrobial activity. Importantly, antibacterial effects cannot necessarily be attributed to individual major constituents. Pieracci et al. [45] showed that hemp EOs were more active than their main constituent, β-Caryophyllene, tested individually, supporting a contribution of interactions among EO constituents. Consistently, Pellegrini et al. [21] associated the antimicrobial activity of Futura 75 EO with several major terpenes, including β-Caryophyllene, Myrcene, Humulene and α-Pinene, while also suggesting a contribution of minor constituents. Thus, the antibacterial activity of hemp EOs is more appropriately interpreted in relation to their overall chemical profile and potential interactions among constituents than to the abundance of a single compound.

The higher proportion of monoterpenes relative to sesquiterpenes contributes to a lighter and more volatile chemical profile of the EO. Owing to their smaller molecular size and physicochemical properties, monoterpenes may interact differently with biological membranes than the heavier sesquiterpenes, which may contribute to differences in the biological activity of EOs [75,76]. In contrast, β-Caryophyllene, a bicyclic sesquiterpene, demonstrates selective CB2 receptor agonism attributed to its rigid molecular structure, which enables interaction with cannabinoid receptors while avoiding CB1-mediated psychoactive effects [77]. However, the biological activity of complex EOs cannot be directly predicted from the abundance or properties of individual constituents, as interactions among multiple components may substantially influence their overall effects.

Taken together, these findings suggest that the biological activity of hemp EOs is determined not only by the presence of individual monoterpenes, sesquiterpenes, and cannabinoids, but also by their relative proportions and potential synergistic interactions. Most importantly, the present study demonstrates that bracts and leaves remaining after seed harvesting, which represent an underutilized post-harvest biomass, can serve as a source of biologically active EOs. The observed differences between cytotoxic effects toward mammalian cells and antibacterial activity further indicate that greater cytotoxicity does not necessarily correspond to greater antibacterial effect. In the present study, the inflorescence-derived UFL and LFL oils, which were dominated by sesquiterpenes and contained substantially higher proportions of (Z)-Caryophyllene, showed greater cytotoxic and haemolytic effects but generally lower antibacterial activity than the infructescence-derived UFR and LFR oils. This apparent difference may reflect the fundamentally different organisation and physicochemical properties of mammalian plasma membranes and bacterial cell envelopes. In bacteria, the peptidoglycan layer and, in Gram-negative species, the additional outer membrane may restrict the accessibility of EO constituents to the cytoplasmic membrane, while differences in membrane lipid composition and surface properties may further influence susceptibility. In addition, interactions among EO constituents may result in different effects on mammalian and bacterial membranes [78,79,80]. Thus, the contrasting cytotoxic and antibacterial profiles of the tested EOs are likely to reflect their overall chemical composition and the distinct structural properties of their cellular targets rather than the abundance of a single constituent. The combination of antibacterial activity with comparatively low cytotoxicity and haemolytic activity observed for UFR and particularly LFR supports further investigation of infructescence-derived EOs in application-oriented antimicrobial studies.

## 3. Materials and Methods

### 3.1. Experimental Conditions

The experiment was set up on an agricultural field located in the administrative district of Piekary Śląskie, southern Poland (50°21′19″ N; 19°00′17″ E). The soil was characterized by a silty loam texture, a slightly alkaline pH (7.17 ± 0.4 in KCl), a total nitrogen content of 1.89±0.39 g kg^−1^, and a total carbon content of 32 g kg^−1^, of which approximately 3% was organic carbon. The available phosphorus and potassium contents were 9.73 mg P_2_O_5_ 100 g^−1^ and 24.43 mg K_2_O 100 g^−1^. Three 8 m × 8 m plots were established in the experimental field.

Seeds of hemp (*Cannabis sativa* L. subsp. *sativa* cv. Futura 75, purchased from MODERIA PPHU, Poland, www.moderia.pl; certificate number: F1545A047522) were sown manually at a depth of 3–4 cm during the last week of May 2023. The distance between rows was 50 cm, and the spacing between plants within each row was 10 cm. The plots were hand-weeded twice during the growth season, and no herbicides or pesticides were applied.

### 3.2. Raw Material Collection

The inflorescences and infructescences were manually harvested from randomly selected plants from each plot at the phenological stage corresponding to the beginning of flowering (Code 2201) and the end of seed maturity (95% of seeds hard; Code 2205), respectively [81]. The collected inflorescences and infructescences (approximately 0.8–1.2 m in length) were divided into two equal parts—upper and lower. Stems and lateral branches were removed from all samples, whereas seeds were additionally removed from the infructescences. For each plot, four groups of raw material were distinguished: UFL—flowers, bracts, and leaves of the upper part of the inflorescence; LFL—flowers, bracts, and leaves of the lower part of the inflorescence; UFR—bracts and leaves of the upper part of the infructescence; LFR—bracts and leaves of the lower part of the infructescence. The raw material was carefully placed in plastic bags and transported to the laboratory, where it was air-dried at ambient temperature (21 °C) in the dark to minimize photo-oxidation (aerial dry weight, ADW).

### 3.3. Qualitative and Quantitative Analysis of Essential Oil

#### 3.3.1. Essential Oil Isolation

The air-dried plant material was mechanically comminuted immediately before distillation. For each independent isolation, 30.0 g of plant material was mixed with 400 mL of distilled water, corresponding to a plant material-to-water ratio of 1:13.3 (*w*/*v*), and subjected to hydrodistillation for 3 h using a standard Deryng-type apparatus. For each type of plant material, samples originating from the three independent field plots were hydrodistilled separately, resulting in three independent EO samples (*n* = 3). The EOs were collected over water, separated, dried over anhydrous sodium sulfate, transferred to amber vials, and stored at 4 °C in the dark until chromatographic analysis. A representative subsample of each type of raw material was weighed before and after being additionally dried in a forced ventilation oven for 3 h at 105 °C to determine the dry weight (DW). EO yield was expressed as a percentage of plant dry mass and presented as mean ± SD (*n* = 3).

#### 3.3.2. Chromatographic Analyses

For each plant-material type, the EOs obtained from the three independent hydrodistillations were pooled prior to chromatographic analysis. Each pooled EO sample was then analysed in triplicate under identical chromatographic conditions according to procedures described previously [82] using gas chromatography combined with a triple quadrupole mass spectrometry system (GC–MS/MS Varian 4000 system, Varian, Palo Alto, CA, USA). The compounds were separated on a 30 m × 0.25 mm × 0.25 μm VF–5 ms column (Varian, USA). The column temperature was increased from 50 °C to 250 °C at a rate of 4 °C min^−1^. The injector temperature was 250 °C; the split ratio was 1:50, and the injection volume was 5 μL. The MS parameters were as follows: electron ionisation (EI) mode with ionization voltage of 70 eV, ion source temperature of 200 °C, and a scan range of 40–870 Da. Gas chromatography with flame ionization detection (GC-FID; Varian 3800 system, Varian, USA) was subsequently used for quantitative EO analysis. The compounds were separated on a 30 m × 0.25 mm × 0.25 μm DB-5 column (J&W Scientific, Folsom, CA, USA). The GC program was identical to that used for GC–MS analysis.

#### 3.3.3. Qualitative and Quantitative Analysis

Compound assignments were based on complementary mass-spectrometric and chromatographic criteria. Experimental retention indices (RIexp) were calculated according to the van den Dool–Kratz [83] approach using a homologous series of n-alkanes (C8–C28) analysed under the same chromatographic conditions. The RIexp values were compared with published values for non-polar stationary phases and with retention-index databases. Electron-ionisation mass spectra were matched against the NIST library [84] and relevant literature data [44,82,85,86]. A library similarity of at least approximately 85% and a difference between experimental and reference RI values not exceeding approximately 15 index units were used as operational acceptance criteria, and diagnostic ions and fragmentation patterns were additionally inspected. Because no analyte-specific authentic standards were analysed, all compounds are reported as tentatively identified. Relative contents were calculated from GC–FID peak areas using n-alkanes C12 and C19 as internal standards, without compound-specific response-factor correction.

### 3.4. Cell Culture Experiments

Normal human skin fibroblasts (BJ human skin fibroblast cell line, CRL-2522™, ATCC, Manassas, VA, USA) were maintained in Earle’s Minimum Essential Medium (EMEM, ATCC) supplemented with 10% fetal bovine serum (PAN Biotech, Aidenbach, Germany) and 1% antibiotic solution containing penicillin (100 U mL^−1^) and streptomycin (1000 µg mL^−1^) (Sigma-Aldrich Chemicals, Warsaw, Poland). Cells were cultured under standard conditions at 37 °C in a humidified incubator with 5% CO_2_ and 95% air.

#### 3.4.1. Preparation of Essential Oil Solutions for Cell Culture Experiments

Stock solutions of the essential oils (EOs) were prepared by dissolving 100 mg of each EO in 1 mL of dimethyl sulfoxide (DMSO; Sigma-Aldrich, St. Louis, MO, USA), yielding a stock concentration of 100 mg mL^−1^. The stock solutions were subsequently diluted in cell culture medium, and two-fold serial dilutions were prepared to obtain final EO concentrations ranging from 500 to 0.97 µg mL^−1^. At the highest tested EO concentration of 500 µg mL^−1^, the final DMSO concentration was 0.5% (*v*/*v*) and decreased proportionally in the subsequent serial dilutions. A solvent control containing culture medium supplemented with 0.5% (*v*/*v*) DMSO, without the tested EOs, was included to assess the potential effect of the solvent on cell viability. No reduction in cell viability was observed in the solvent control compared with untreated cells. The maximum DMSO concentration was selected based on previous reports indicating that 0.5% (*v*/*v*) DMSO can be tolerated by cultured mammalian cells under defined experimental conditions [87].

#### 3.4.2. Cytotoxicity Assay

The cytotoxicity of the tested EOs was evaluated using the MTT assay in accordance with ISO Standard No. 10993-5:2009 [88], following the protocol described in our previous study by Sugier et al. [89]. Briefly, BJ human skin fibroblasts were exposed to the tested EOs at concentrations ranging from 0.97 to 500 µg mL^−1^ for 24 and 48 h. After incubation, cellular metabolic activity was assessed using the MTT assay, and absorbance was measured spectrophotometrically at 570 nm. The MTT signal was expressed as a percentage relative to the untreated control, which was set at 100%. Each experiment was performed in triplicate (*n* = 3 technical replicates).

#### 3.4.3. Live/Dead Cell Viability Assay

For the qualitative assessment of cytocompatibility, Live/Dead fluorescence staining was performed following the protocol described in our previous study [89], with minor modifications. BJ fibroblasts were seeded under the same conditions as those used for the MTT assay and, after attachment, exposed to the tested EOs for 24 h. Three representative concentrations were selected for microscopic analysis: 0.97, 31.25, and 500 µg mL^−1^, corresponding to the lowest, intermediate, and highest concentrations tested in the cytotoxicity assay. After incubation, the cells were stained using a Live/Dead double-staining kit (Sigma-Aldrich Chemicals, Warsaw, Poland) according to the manufacturer’s instructions and observed using a confocal laser scanning microscope (CLSM; Olympus FluoView equipped with FV1000, Shinjuku, Japan). Live cells were visualized as green fluorescent cells, whereas dead cells were identified by red fluorescence. The experiment was performed in three independent replicates (*n* = 3). Multiple microscopic fields were examined for each experimental condition, and representative images reflecting the staining pattern consistently observed across the independent experiments were selected for presentation. All images were acquired using identical microscope settings, including laser power, detector gain, magnification, and image-processing parameters.

#### 3.4.4. WST-8 Assay

The effect of the tested EOs on the metabolic activity of BJ human skin fibroblasts was evaluated using the water-soluble tetrazolium salt (WST-8) assay. BJ fibroblasts were seeded in 96-well plates at a density of 3 × 10^4^ cells mL^−1^ and allowed to attach for 24 h. Subsequently, the cells were exposed to the tested EO extracts at three representative concentrations: 0.97, 31.25, and 500 µg mL^−1^. The cells were cultured for 4 or 6 days, and on day 4, the medium containing the tested EOs was replaced with freshly prepared medium containing corresponding EO concentrations. At the end of each incubation period, cellular metabolic activity was assessed using the WST-8 assay (Sigma-Aldrich) according to the manufacturer’s instructions. Absorbance was measured as optical density at 450 nm (OD450) using a microplate reader. The obtained OD450 values were used as a measure of tertrazolium reduction relative to the control.

Following completion of the quantitative assay, the cells were fixed according to the procedure described by Nurzynska et al. [90]. Briefly, the cells were fixed with 3.7% formaldehyde to preserve their morphology and cellular architecture. Subsequently, the samples were treated with 0.2% Triton X-100 to permeabilize the cell membranes, enabling efficient penetration of fluorescent probes during subsequent staining procedures.

#### 3.4.5. Immunofluorescence Staining of Cell Nuclei, Cytoskeleton, and Collagen Type I

After completion of the WST-8 assay, previously fixed BJ fibroblasts were used for immunofluorescence staining to evaluate cell nuclei, cytoskeletal organization, and the distribution of collagen type I after 4 days of incubation with the tested EOs. Non-specific binding sites were blocked with 1% bovine serum albumin (BSA) for 30 min at room temperature. After removal of the blocking solution, the cells were incubated overnight (16 h) at 4 °C with a rabbit recombinant monoclonal anti-collagen type I primary antibody (Invitrogen). On the following day, the samples were incubated with a goat anti-rabbit secondary antibody conjugated with Alexa Fluor 488 to visualize collagen type I (green fluorescence). F-actin was stained with Alexa Fluor 635 to visualize the cytoskeleton (red fluorescence), whereas cell nuclei were counterstained with Hoechst 33342 (blue fluorescence). The stained cells were observed using a confocal laser scanning microscope (CLSM; Olympus FluoView equipped with FV1000, Shinjuku, Japan). The experiment was performed in three independent replicates (*n* = 3). Multiple microscopic fields were examined for each experimental condition, and representative images reflecting the staining pattern consistently observed across the independent experiments were selected for presentation. All images were acquired using identical microscope settings, including laser power, detector gain, magnification, and image-processing parameters.

### 3.5. Haemolysis Assay

The haemolytic activity of the tested EOs was evaluated using citrated human blood collected from a single healthy volunteer, with the approval of the Bioethics Committee of the Medical University of Lublin (approval no. KB-0024/19/02/2025). Stock solutions of the EOs were prepared by dissolving 100 mg of each EO in 1 mL of dimethyl sulfoxide (DMSO; Sigma-Aldrich, St. Louis, MO, USA), yielding a stock concentration of 100 mg mL^−1^. The stock solutions were subsequently diluted in phosphate-buffered saline (PBS) to prepare working solutions at twice the intended final concentrations, so that mixing 1 mL of each EO solution with 1 mL of diluted citrated blood resulted in final EO concentrations ranging from 0.97 to 500 µg mL^−1^. At the highest tested EO concentration, the final DMSO concentration in the assay mixture was 0.5% (*v*/*v*). For the assay, 1 mL of each EO working solution was mixed with 1 mL of appropriately diluted citrated blood and incubated for 3 h at 37 °C under gentle agitation. After incubation, the samples were centrifuged, and the supernatants were collected. Drabkin’s reagent (Chempur, Piekary Śląskie, Poland) was then added to each supernatant, followed by incubation for 15 min at 37 °C. Absorbance was measured at 540 nm using a microplate reader. Blood incubated without the tested EO extracts served as the negative control, whereas blood treated with 0.1% Triton X-100 was used as the positive control. The percentage of haemolysis was calculated relative to the negative and positive controls. Each experiment was performed in quadruplicate (*n* = 4 technical replicates).

### 3.6. Blood Clotting Time Assay

The effect of the tested EOs on blood clotting time was evaluated using human blood collected from a healthy volunteer, with the approval of the Bioethics Committee of the Medical University of Lublin (approval no. KB-0024/19/02/2025). Before the experiment, the blood sample was maintained at 37 °C. Stock solutions of the EOs were prepared by dissolving 100 mg of each EO in 1 mL of dimethyl sulfoxide (DMSO; Sigma-Aldrich, St. Louis, MO, USA), yielding a concentration of 100 mg mL^−1^. The stock solutions were subsequently diluted to prepare working solutions at concentrations selected to obtain final EO concentrations of 0.97, 31.25, and 500 µg mL^−1^ after mixing with blood. At the highest tested EO concentration, the final DMSO concentration in the assay mixture was 0.5% (*v*/*v*). Blood coagulation was initiated by the addition of CaCl_2_, and timing was started immediately after mixing the blood with the tested EO solutions. The tubes were gently inverted every 10 s until a stable blood clot had formed. Blood clotting time in the presence of the tested EOs was compared with the clotting time of blood activated with CaCl_2_ in the absence of the tested EOs. Each experiment was performed in triplicate (*n* = 3 technical replicates).

### 3.7. Microorganisms and Growth Conditions

A collection of reference microbial strains comprising Gram-positive, Gram-negative, and microaerophilic or aerobic bacteria was used in the study, including *Staphylococcus aureus* ATCC 25923, *Staphylococcus epidermidis* ATCC 12228, *Enterococcus faecalis* PCM 896 (EF1) and a wild clinical isolate (EF2, w.c.i.), *Escherichia coli* ATCC 25922, and *Pseudomonas aeruginosa* ATCC 27853. In addition, microaerophilic strains included oral streptococci *Streptococcus mutans* PCM 2020 and *Streptococcus sanguinis* PCM 2335, as well as *Cutibacterium acnes* ATCC 11827, PCM 2334, and PCM 2400 (PCM 2334 and PCM 2400 were obtained from the Polish Collection of Microorganisms under the former designation *Propionibacterium acnes*).

Aerobic bacteria were cultured on Mueller–Hinton agar and in Mueller–Hinton broth (Oxoid, Basingstoke, UK) and incubated at 37 °C for 24 h under aerobic conditions, while microaerophilic strains were grown on Brain Heart Infusion (BHI) medium (BioMaxima, Lublin, Poland) and incubated at 37 °C for 48 h under anaerobic conditions. Prior to the experiments, all inocula were standardized to 0.5 McFarland (~1.5 × 10^8^ CFU mL^−1^), and all assays were performed in triplicate under aseptic conditions.

### 3.8. Agar Disk Diffusion Assay

Preliminary antimicrobial screening of the EOs was carried out using the agar disk diffusion method as described by Bauer et al. [91], with minor modifications. Standardized microbial inocula (0.5 McFarland) were evenly spread onto agar surfaces using sterile swabs.

The essential oils were dissolved in dimethyl sulfoxide (DMSO) to obtain stock solutions at a concentration of 10 mg mL^−1^. Before application onto the agar discs, the solutions were thoroughly vortexed to ensure complete homogenization. For assay validation, sparfloxacin (CAS No. 110871-86-8; purity ≥ 98%; Pol-Aura, Olsztyn, Poland), at 100 µg per application, prepared from analytical reference standards, was included as a positive control and tested under the same experimental conditions as the EOs. Subsequently, 10 µL of each EO solution (equivalent to 100 µg per disc) was applied onto the inoculated agar plates. After incubation under organism-specific conditions, the inhibition zones were measured using a calibrated digital caliper. Antimicrobial activity was expressed as the diameter of growth inhibition zones (mm).

### 3.9. Determination of Minimum Inhibitory Concentration (MIC)

MIC values were determined using the broth microdilution method in sterile 96-well microtiter plates. Appropriate culture media (Mueller–Hinton broth for aerobic bacteria and BHI broth for microaerophilic strains) were dispensed into wells prior to inoculation.

The EO extracts were dissolved in dimethyl sulfoxide (DMSO) to obtain stock solutions at a concentration of 10 mg mL^−1^ and thoroughly vortexed before use. Two-fold serial dilutions of the tested EO extracts were prepared directly in the microplates, resulting in final concentrations ranging from 1.6 to 0.00625 mg mL^−1^. Each dilution step was homogenized by pipetting to ensure uniform dispersion of the essential oils in the culture medium. Each well contained a final bacterial inoculum of approximately 1.5 × 10^6^ CFU mL^−1^, obtained by adding 2 µL of a 0.5 McFarland bacterial suspension (≈1.5 × 10^8^ CFU mL^−1^) to a final well volume of 200 µL.

The experimental design included a growth control, a sterility control, and a solvent control to account for potential effects of DMSO and essential oil coloration. A corresponding DMSO solvent control was included and confirmed that the tested DMSO did not affect bacterial growth under the applied experimental conditions. The reference antibiotic sparfloxacin was included as a positive control and tested in parallel with the essential oils using the same broth microdilution procedure. Plates were incubated under appropriate conditions. Reagent control wells containing the corresponding concentrations of essential oils in the culture medium without microbial inoculum were included to exclude interference from the intrinsic turbidity or color of the samples during MIC determination. The MIC was defined as the lowest concentration of the EO that completely inhibited visible microbial growth.

### 3.10. Statistical Analysis

One-way ANOVA followed by Dunnett’s post hoc test was used to analyse the cytotoxic activity of the hemp EOs, their haemolytic activity, WST-8 assay results, and the effect of EOs on blood clotting time, using GraphPad Prism 8.0.0 (GraphPad, La Jolla, CA, USA). Two-way ANOVA followed by Tukey’s post hoc test was applied to assess the effects of raw-material type, position, and their interaction on EO yield, and the effects of EO type and bacterial strain on the bacterial growth inhibition zones, using Statistica 6.0 (Stat. Soft, Inc., Kraków, Poland). Differences were considered statistically significant at *p* < 0.05. Principal component analysis (PCA) was performed to evaluate the relationships between the chemical composition of the examined EOs and bacterial growth inhibition zones. The analysis included EO components with a content greater than 7% and the studied bacterial growth inhibition zones. Prior to PCA, the data were centred and standardized. Statistical analyses were carried out using MVSP version 3.1 [92].

## 4. Conclusions

The present study demonstrated that the essential oils (EOs) of hemp (*Cannabis sativa* L. cv. Futura 75) possess a chemical profile characteristic of this cultivar, while exhibiting greater compositional diversity under the climatic and soil conditions of Central Europe than previously reported for the same cultivar grown in other regions. This chemical variability was accompanied by differences in the biological activity of the EOs, suggesting that both the origin of the plant material and the quantitative relationships among their constituents contribute to their functional properties.

Most importantly, the results demonstrated that seed-depleted infructescences, representing an underutilized post-harvest by-product biomass, constitute a valuable source of biologically active EOs. Essential oils obtained from these materials displayed the most favourable biological safety profile, characterized by low cytotoxicity toward BJ human skin fibroblasts, minimal haemolytic activity, and no significant effects on blood coagulation. In contrast, EOs derived from inflorescences harvested at the initial flowering stage showed greater cytotoxic and haemolytic potential, indicating a stronger interaction of their constituents with cellular membranes.

The antimicrobial activity of the tested EOs depended primarily on the type of plant material rather than on the taxonomic affiliation of the bacterial strains, with infructescence-derived EOs demonstrating superior efficacy against most tested microorganisms. Their particularly promising activity against *Cutibacterium acnes*, together with the observed safety profile, supports further investigation of their potential use in topical antimicrobial formulations for dermatological and cosmetic applications. Furthermore, their antibacterial activity against oral pathogens provides a rationale for future investigations into their activity against cariogenic biofilms, bacterial adhesion, and multispecies oral microbiota.

Overall, the present findings indicate that the valorisation of seed-depleted infructescences may contribute to a more sustainable utilization of hemp biomass by transforming an underutilized post-harvest residue into a valuable source of biologically active EOs with potential for further development in pharmaceutical, cosmetic, and oral healthcare contexts. Future studies could also explore solvent extracts obtained from seed-depleted infructescences to complement the present findings and achieve a more comprehensive valorisation of this underutilized plant material, particularly with respect to the biological potential of its non-volatile constituents.

## Figures and Tables

**Table 1:** Essential oil content in hemp inflorescences and seed-depleted infructescences. UFL—flowers, bracts and leaves from the upper part of the inflorescence, LFL—flowers, bracts and leaves from the lower part of the inflorescence, UFR—bracts and leaves from the upper part of the infructescence, LFR—bracts and leaves from the lower part of the infructescence. Values are expressed as mean ± SD (*n* = 3). Values within a row marked with different letters are significantly different (Tukey’s test, *p* < 0.05).

	UFL	LFL	UFR	LFR
EO content (% DM)	0.288 ^ab^ ± 0.010	0.265 ^b^ ± 0.009	0.310 ^a^ ± 0.012	0.272 ^b^ ± 0.011

**Table 3:** Minimum inhibitory concentration (MIC) [mg mL^−1^] of hemp essential oils against the tested bacterial strains. SA—*S. aureus* ATCC 25923; SE—*S. epidermidis* ATCC 12228; EF1—*E. faecalis* PCM 896; EF2—*E. faecalis* (w.c.i.); EC—*E. coli* ATCC 25922; PA—*P. aeruginosa* ATCC 27853; CA—*C. acnes* ATCC 11827; PAC1—*C. acnes* PCM 2334; PAC2—*C. acnes* PCM 2400; SM—*S. mutans* PCM 2020; SS—*S. sanguinis* PCM 2335; UFL—flowers, bracts and leaves from the upper part of the inflorescence; LFL—flowers, bracts and leaves from the lower part of the inflorescence; UFR—bracts and leaves from the upper part of the infructescence; LFR—bracts and leaves from the lower part of the infructescence.

MIC [mg mL^−1^]											
	SA	SE	EF1	EF2	EC	PA	CA	PAC1	PAC2	SM	SS
UFL	0.10	0.05	0.05	0.20	>1.6	>1.6	0.40	0.40	0.80	1.6	>1.6
LFL	1.60	0.40	>1.6	>1.6	>1.6	>1.6	0.80	0.40	0.80	>1.6	>1.6
UFR	0.10	0.05	0.10	0.20	0.20	0.40	0.10	0.10	0.10	0.10	0.05
LFR	0.05	0.025	0.05	0.20	0.20	0.20	0.05	0.025	0.05	0.10	0.10
SPARFL	0.00025	0.0005	0.001	0.002	0.00012	0.002	nt	0.0005	0.001	0.001	0.001

MIC values > 1.6 mg mL^−1^ indicate that no inhibitory effect was observed within the tested concentration range; SPARFL—sparfloxacin; nt—not tested.

**Table 4:** Results of PCA based on the 7 main compounds of the *C. sativa* essential oils. (a) Eigenvalues and variance (%) explained by the first two PCA axes; (b) Loading components for each variable associated with the two axes. SA—*S. aureus* ATCC 25923, SE—*S. epidermidis* ATCC 12228, EF1—*E. faecalis* PCM 896, EF2—*E. faecalis* (w.c.i.), EC—*E. coli* ATCC 25922, PA—*P. aeruginosa* ATCC 27853, CA—*C. acnes* ATCC 11827, PAC1—*C. acnes* PCM 2334, PAC2—*C. acnes* PCM 2400. SM—*S. mutans* PCM 2020, SS—*S. sanguinis* PCM 2335.

Chemical Variables	Axis 1	Axis 2	Axis 3
(a)			
Eigenvalues	13.686	2.413	1.437
Percentage	76.03	13.41	7.98
Cum. percentage	76.03	89.44	97.42
(b)			
α-Pinene	0.262	−0.11	−0.147
Myrcene	0.25	−0.17	−0.227
(Z)-Caryophyllene	−0.253	−0.168	0.195
(E)-Caryophyllene	0.26	−0.158	−0.096
(E)-β-Farnesene	−0.252	0.226	−0.059
Caryophyllene oxide	−0.106	0.589	−0.046
Cannabidiol	−0.135	0.295	0.602
SA	0.218	0.324	−0.216
SE	0.221	0.316	0.083
EF1	0.198	0.361	−0.297
EF2	0.258	0.127	−0.148
EC	0.241	−0.132	0.301
PA	0.262	−0.006	0.122
CA	0.245	0.177	0.222
PAC1	0.252	0.066	0.241
PAC2	0.257	−0.093	0.033
SM	0.249	0.013	0.285
SS	0.254	−0.086	0.229

## Data Availability

The data can be accessed at the following link: https://drive.google.com/drive/folders/1-p-dEyXxmretDmP1K2m2TWvzAVkAUXHs?usp=drive_link (accessed on 16 August 2026). We confirm that the repository contains the complete set of data underlying the results presented in this article.
